# Application of Six Detection Methods for Analysis of Paralytic Shellfish Toxins in Shellfish from Four Regions within Latin America

**DOI:** 10.3390/md18120616

**Published:** 2020-12-03

**Authors:** Andrew D. Turner, Sophie Tarnovius, Robert G. Hatfield, Mickael Teixeira Alves, Maggie Broadwater, Frances Van Dolah, Ernesto Garcia-Mendoza, Dinorah Medina, Maria Salhi, Alejandra B. Goya, Fernanda Barrera, Daniel Carrasco, Ignacio Rubilar, Benjamin A. Suarez-Isla

**Affiliations:** 1 Centre for Environment, Fisheries and Aquaculture Science (Cefas), Barrack Road, The Nothe, Weymouth, Dorset DT4 8UB, UK; sophie.tarnovius@gmx.de (S.T.); Robert.Hatfield@Cefas.co.uk (R.G.H.); Mickael.teixeiraalves@cefas.co.uk (M.T.A.); 2 Technische Universität München, Walther-Meißner-Straße 3, 85748 Garching, Germany; 3 National Oceanic and Atmospheric Administration, National Centers for Coastal Ocean Science Stressor Detection and Impacts Division, Charleston, SC 29412, USA; maggie.broadwater@noaa.gov (M.B.); franvandolah@gmail.com (F.V.D.); 4 Departamento de Oceanografía Biológica, Centro de Investigación Científica y de Educación Superior de Ensenada. Carr. Ens-Tij 3608, Ensenada, Baja California 22860, Mexico; ergarcia@cicese.mx; 5 Dirección Nacional de Recursos Acuáticos, PO Box 1612, Constituyente 1497, Montevideo 11200, Uruguay; dmedina@mgap.gub.uy (D.M.); msalhi@mgap.gub.uy (M.S.); 6 Marine Biotoxin Department, Mar del Plata Regional Laboratory, Agri-food Health and Quality National Service (Senasa), Mar del Plata 7600, Argentina; agoya@senasa.gov.ar; 7 Laboratory of Marine Toxins, Institute of Biomedical Sciences, Faculty of Medicine, University of Chile, Santiago 8320000, Chile; mferbarr@uchile.cl (F.B.); dcarrasco@uchile.cl (D.C.); irubilar@med.uchile.cl (I.R.); bsuarez@u.uchile.cl (B.A.S.-I.)

**Keywords:** paralytic shellfish poisoning (PSP), LC-FLD, LC-MS/MS, MBA, RBA, toxin profiles

## Abstract

With the move away from use of mouse bioassay (MBA) to test bivalve mollusc shellfish for paralytic shellfish poisoning (PSP) toxins, countries around the world are having to adopt non-animal-based alternatives that fulfil ethical and legal requirements. Various assays have been developed which have been subjected to single-laboratory and multi-laboratory validation studies, gaining acceptance as official methods of analysis and approval for use in some countries as official control testing methods. The majority of validation studies conducted to date do not, however, incorporate shellfish species sourced from Latin America. Consequently, this study sought to investigate the performance of five alternative PSP testing methods together with the MBA, comparing the PSP toxin data generated both qualitatively and quantitatively. The methods included a receptor binding assay (RBA), two liquid chromatography with fluorescence detection (LC-FLD) methods including both pre-column and post-column oxidation, liquid chromatography with tandem mass spectrometry (LC-MS/MS) and a commercial lateral flow assay (LFA) from Scotia. A total of three hundred and forty-nine shellfish samples from Argentina, Mexico, Chile and Uruguay were assessed. For the majority of samples, qualitative results compared well between methods. Good statistical correlations were demonstrated between the majority of quantitative results, with a notably excellent correlation between the current EU reference method using pre-column oxidation LC-FLD and LC-MS/MS. The LFA showed great potential for qualitative determination of PSP toxins, although the findings of high numbers of false-positive results and two false negatives highlighted that some caution is still needed when interpreting results. This study demonstrated that effective replacement methods are available for countries that no longer wish to use the MBA, but highlighted the importance of comparing toxin data from the replacement method using local shellfish species of concern before implementing new methods in official control testing programs.

## 1. Introduction

**Figure 1:**
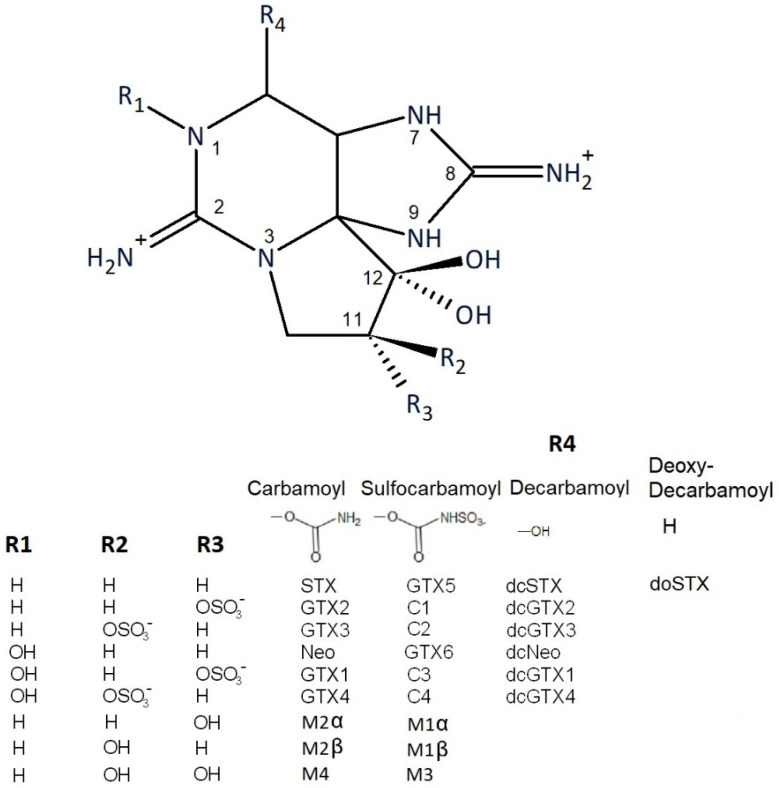
Structures of saxitoxin analogues incorporated into testing methods from this study.

Paralytic shellfish toxins (PSTs) are harmful neurotoxins originating from phytoplankton of the genera *Gymnodinium*, *Pyrodinium* and *Alexandrium* that periodically accumulate in shellfish through filter feeding. These may result in sickness and even fatalities following human consumption of contaminated shellfish products [1,2]. The toxins are members of the saxitoxin family, which contain over 55 structurally related compounds [3] (Figure 1). Toxicity relates to the action of the toxins on voltage-gated sodium channels, leading to numbness, tingling sensations and nausea, with high doses causing paralysis and death by asphyxiation [4,5]. The hydrophilic saxitoxins consist of three different groups of compounds, as classified by their chemical structure: *N*-sulfocarbamoyl, decarbamoyl and the carbamoyl toxins [6]. Other hydrophobic congeners are also known to exist, most notably in bivalves exposed to *Gymnodinium catenatum* [7], including those from South America [8].

Occurrences of PSP-producing harmful algal blooms have been recognised along the Pacific and Atlantic coasts of Latin America (LA) for many years [9,10,11,12,13,14]. In Argentina, *Gymnodinium catenatum* was initially recorded in 1961-1962 around Mar del Plata [15] and currently occurs in the northern regions of the country. *Alexandrium catenella* (formerly *A. tamarense*) was identified as the cause of PSP outbreaks in 1980 and has been recorded annually from southern Argentina up to the coast of Uruguay [16,17,18,19,20]. Intense blooms of *A. catenella* have also been measured within the Beagle Channel in southern Argentina [21,22,23]. Further up the Atlantic coast in Uruguay, repeated blooms of *A. catenella* and *G. catenatum* have been identified regularly since 1991 and 1992, respectively [24,25,26], with the subsequent identification of *A. fraterculus* [9]. Harmful algal blooms also severely impact the Pacific coastal regions of North and Central America [12,27,28], where *G. catenatum* and *Pyrodinium bahamense* var. *compressum* are typically associated with outbreaks [29,30,31,32]. *Alexandrium* species including *A. catenella* have also been detected in Mexico [32,33,34], although none associated with shellfish contamination [35,36]. In Chile, *A. catenella* has been reported since the 1970s [9,11]. HABs of *A. catenella* have increased in their frequency, extension, duration and intensity [37,38].

High levels of PSTs in Latin American shellfish have resulted in a significant number of PSP outbreaks. PSTs have been measured in a wide range of bivalve molluscs as well as other marine species including gastropods [39,40,41,42,43,44]. During 1980 in Argentina, the PSP toxicity of mussels was reported at levels equivalent to 312,048 µg STX eq./kg [44,45], with repeated events reported throughout the next 15 years [18,19,46,47,48]. The toxicity of mussels harvested in Patagonia during 1992 was found to reach a maximum of 1,272,000 µg STX eq./kg [21,49]. Consequently, the waters along the Atlantic and Pacific coasts of LA are periodically affected by PST accumulation resulting in human health risks [19,45,50,51,52,53,54,55,56,57,58]. PSTs have also been observed in Uruguayan shellfish, with maximum levels recorded at 82,850 µg STX eq./kg during a 1991 bloom of *A. tamarense* [59,60,61] and at 14,780 µg STX eq./kg following a 1992 bloom of *G. catenatum* [26], Medina, unpublished data]. Human illness due to PSP-contaminated shellfish is also well known along the Pacific coast, with incidents reported as far back as the 1970s in Mexico [62,63] with over 40 deaths and 1200 intoxications recorded between 1976 and 2002 [12,34]. Bivalve species implicated in outbreaks include oysters, clams, mussels and the Geoduck clam, with impacts not only on human shellfish consumers, but also animal and ecosystem health [64]. In Chile, high PSP toxicity has been measured in shellfish since the first outbreak in 1972 in the south, causing intoxications and fatalities [21,48,65,66]. Since then, outbreaks have been found on an annual basis between southern Patagonia and the more northerly regions around Chile [67,68]. Between 1972 and 2004, PSP-contaminated shellfish was responsible for the intoxication of 527 people in Chile, with 32 fatalities [65] with additional impacts including mass mortalities of invertebrates [68] and substantial socio-economic costs [69]. In recent years, blooms of *A. catenella* have been reported to have expanded spatially as well as resulting in catastrophic incidents of human poisonings, including the 2016 “Godzilla-Red tide event” triggered by exceptional El Niño conditions with consequent drastic socio-economic impacts [70,71].

To ensure consumer protection, monitoring of toxic phytoplankton and PSTs in shellfish is a statutory requirement, including those countries exporting shellfishery products to the EU [72]. The statutory limit for PSTs in flesh is 800 µg saxitoxin equivalents (STX eq.) per kg of shellfish flesh [73] as described by EC Regulation 853/2004. For many years, the official reference method in the EU and LA for detecting PSTs has been the PSP mouse bioassay (MBA) [74,75]. The method has provided a useful quantitative monitoring tool, although the method is known to be affected by low sensitivity, poor reproducibility and is subject to matrix interferences [5,76]. In recent years, alternative chemical or biomolecular methods have been tested and validated for PST detection. In 2006, a pre-column oxidation (PreCOX) liquid chromatography with fluorescence detection (LC-FLD) [73,77,78,79] method was validated and accepted as an alternative method of analysis for official control testing within the EU (Regulation EC 2074/2005 as amended) [80] and since 1 January 2019 has become the EU reference method [81,82].

Similarly, a post-column oxidation (PCOX) LC-FLD method was validated [83,84] and both LC-FLD methods have been adopted by the AOAC as Official First Action methods (AOAC 2005.06 [74] and AOAC 2011.02 [85]). The PreCOX method is implemented into the official control testing programmes of European member states, the UK and New Zealand [82,86,87,88,89] with the PCOX method approved for use in the US and Canada by the Interstate Shellfish Sanitation Conference [90]. In 2011, a PSP receptor binding assay (RBA) was validated [91,92] and adopted by the AOAC as a first action method (AOAC 2011.27 [93]) for the analysis of mussels and clams. More recently, a method using hydrophilic interaction liquid chromatography with tandem quadrupole mass spectrometry (HILIC-MS/MS) has been developed and validated for PST testing in 12 different shellfish species [94,95] and has undergone successful interlaboratory validation [96], with implementation into regulatory monitoring programmes in New Zealand and Australia. Finally, various commercial antibody-based assays exist which are capable of either qualitative or semi-quantitative determination of PSP in shellfish extracts. One of these, produced by Scotia Rapid Testing Ltd., based upon an immunochromatographic format, is a lateral flow assay (LFA) that has been single-laboratory validated [97] and tested on a range of shellfish samples [98], and is utilised in the US and Mexico under certain scenarios for shellfish screening.

Some of these methods established as alternatives for PSP testing may be used by laboratories in LA for routine official control testing. However, most regions in LA still rely on the MBA. Reasons for this include the considerable efforts and expense required to set-up, validate and implement these alternatives. The choice of method can be confusing as it is also highly dependent on both method performance and the intended export market destination. LA regions intending to export mussels to the US, for example, will be required to use either the MBA, RBA or PCOX LC-FLD method. In addition, within the US legislation, there is the option for use of the Scotia PSP rapid immunochromatographic assay for PSP screening, which offers countries exporting to the US another opportunity for cost-effective toxin testing as part of their regulatory control system. Whilst the EU reference method for PSP is now AOAC2005.06, LA regions have not changed national laws to enforce the implementation of non-animal-based alternative methods.

A wide range of validation studies have been published for PSP testing methods in recent years, but to date very few of these have been focussed on shellfish species typically harvested in LA. Additionally, various authors have assessed the advantages and disadvantages of each of these PSP testing methods, incorporating aspects of sample throughput and turnaround, method performance, financial costs, practicalities and reagent/instrumentation/training requirements [5,82,97,98,99,100,101,102,103,104,105], but again with a primary focus on regions where analytical costs are achievable.

Consequently, there is a need to establish comparative performance between potential regulatory testing methods for PSP utilising shellfish samples harvested from LA in order to evaluate alternative methods to the MBA which are appropriate for the species of relevance in the region. This study therefore evaluated the alternative methods available for monitoring PSTs in shellfish from four different countries within LA. A large range of shellfish species were assessed including mussels, oysters, clams, cockles, scallops and marine gastropods. The total sample toxicities were assessed following quantitation of PSTs using the MBA in comparison with the PreCOX LC-FLD (AOAC 2005.06), the RBA (AOAC 2011.27), the PCOX LC-FLD (AOAC 2011.02), the HILIC-MS/MS [94,95,96] and the LFA manufactured by Scotia Rapid Testing Ltd. (chester, Canada)[97,98].

## 2. Results

### 2.1. Toxicities 

Total PST concentrations were determined by PreCOX, PCOX and LC-MS/MS together with PSP toxicities assessed directly by both MBA and RBA. Table S1 in the Supplementary Materials tabulates all the results obtained for each individual sample. Out of the 349 shellfish samples analysed by PreCOX LC-FLD in this study, total toxicities were found to vary enormously, with 62 samples showing PSP < 16 µg STX eq./kg and toxicities reaching a maximum of >400,000 µg STX eq./kg in a mussel sample originating from Argentina. Table S1 illustrates the high toxicities found in mussels, scallops and snails from Argentina, as well as mussels and clams from Chile, and mussels from Uruguay. In total, 137 samples were found to contain total PSP above the regulatory maximum permitted limit of 800 µg STX eq./kg (39%) as determined by PreCOX LC-FLD.

Analysis of a large number of shellfish exhibiting low or no detectable PST presence enabled the assessment of toxin/toxicity results between multiple methods across a wide geographical extent. In total, 80 samples returned an MBA result of not detected, evidencing either an absence of toxicity or total PST below the MBA LOD of ~320 µg STX eq./kg. All of these samples were analysed by PreCOX, with a mean total PST of 259 µg STX eq./kg, with the results skewed by five samples with PreCOX total PST > 1000 µg STX eq./kg, all from Argentinean mussels and scallops. High toxicities were confirmed in these samples by other methods, showing issues with the original MBA or storage/transportation issues relating to toxin stability issues of the samples, rather than the performance of the non-MBA alternatives (Table S1). Out of these 80 samples, 57 were also analysed by LC-MS/MS, with a mean total PST concentration of 306 µg STX eq./kg, still below the LOD of the MBA. In total, 43 samples returned not detected results following PreCOX, with all of these also showing not detected by MBA and RBA. A total of 39 of these samples were also analysed by LC-MS/MS, with 27 of these (69%) returning total PST < 16 µg STX eq./kg and the remaining positive results reaching a maximum of 200 µg STX eq./kg.

### 2.2. Quantitative Comparison and Toxin Profiles

**Figure 2:**
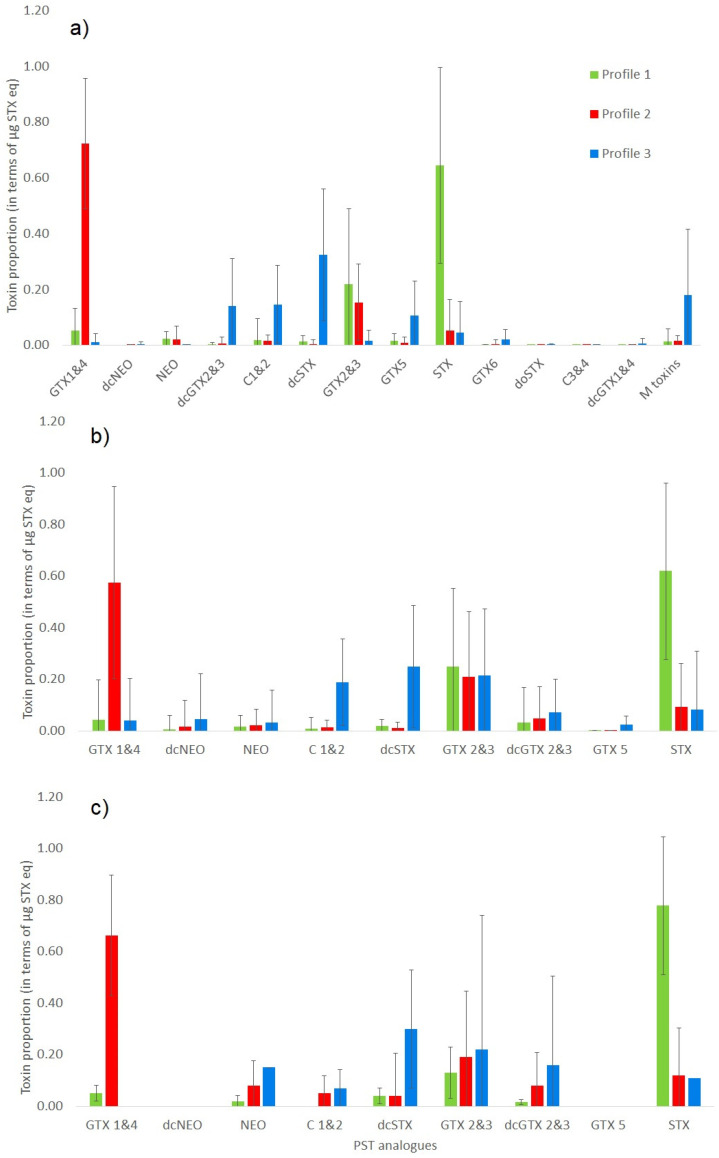
Summary of toxin profiles in terms of saxitoxin equivalents for each of the three profile clusters determined in LA shellfish samples following (**a**) LC-MS/MS analysis, (**b**) PreCOX LC-FLD and (**c**) PCOX LC-FLD.

An initial visual assessment focused on shellfish samples containing total PST concentrations above a 160 µg STX eq./kg threshold, equating to 224 samples run by the EU reference method (PreCOX LC-FLD). Of these samples, 173 returned positive MBA results, 160 PCOX, 199 LC-MS/MS and 100 RBA. A wide variety of toxin profiles were also evidenced across all samples assessed. Table S2 summarises the toxin profile data in terms of saxitoxin equivalents for each sample as determined by LC-MS/MS. The most commonly occurring PST analogue was STX, quantified in 99% of PST-positive shellfish samples. GTX2&3, C1&2, dcSTX, GTX1&4, NEO and GTX5 were also detected in large numbers of samples (85%, 83%, 71%, 70%, 66%, 63%, respectively). K-means clustering analysis highlighted the presence of three main toxin profile types from the dataset, based on the analysis of quantitative data following LC-MS/MS. This method was chosen given its ability to quantify the largest number of PST analogues, with each epimeric pair quantified separately. Figure 2a illustrates the mean profiles, showing profile 1 to be dominated by STX (65 ± 35%) followed by GTX2&3 (22 ± 27%), with low/trace relative concentrations of GTX1&4, NEO, C1&2 and GTX5 (all < 2% each). On the other hand, profile 2 showed a near total presence of gonyautoxins, with 72 ± 23% GTX1&4, 15 ± 14% GTX2&3 and just 5 ± 11% STX, with low/trace proportions of NEO, dcGTX2&3, C1&2, GTX5 and M toxins (Table 1) (all <2% each). Finally, cluster profile 3 represented a larger mix of toxin analogues, with no clear dominance of any one toxin and mean proportions showing significant presence of other less-commonly encountered toxins. Mean profiles included most notably 32 ± 24% dcSTX, 15 ± 14% C1&2, 14 ± 17% dcGTX2&3 and 11 ± 12% GTX5. Profile 3 also incorporated higher relative proportions of M toxins, with a total mean proportion of 18 ± 24% for M1-4 summed. Low/trace proportions of GTX6, dcGTX1&4, GTX1&4 and GTX2&3 were also detected (<2% each). Supplemental Figure S1 illustrates the three profile types in relation to both country of origin and shellfish species. Samples exhibiting cluster 1 were found only in Argentina and Chile, with cluster 2 samples found to dominate Argentinean samples as well as Uruguay and Chile. All Mexican geoduck samples were associated with cluster 3 profile. Overall, mussels were most commonly associated with cluster 2, with the majority of gastropods exhibiting profile 1. No other clear patterns were evident from the distribution of data shown in Figure S1.

#### 2.2.1. Comparison of Five Quantitative Methods

**Figure 3:**
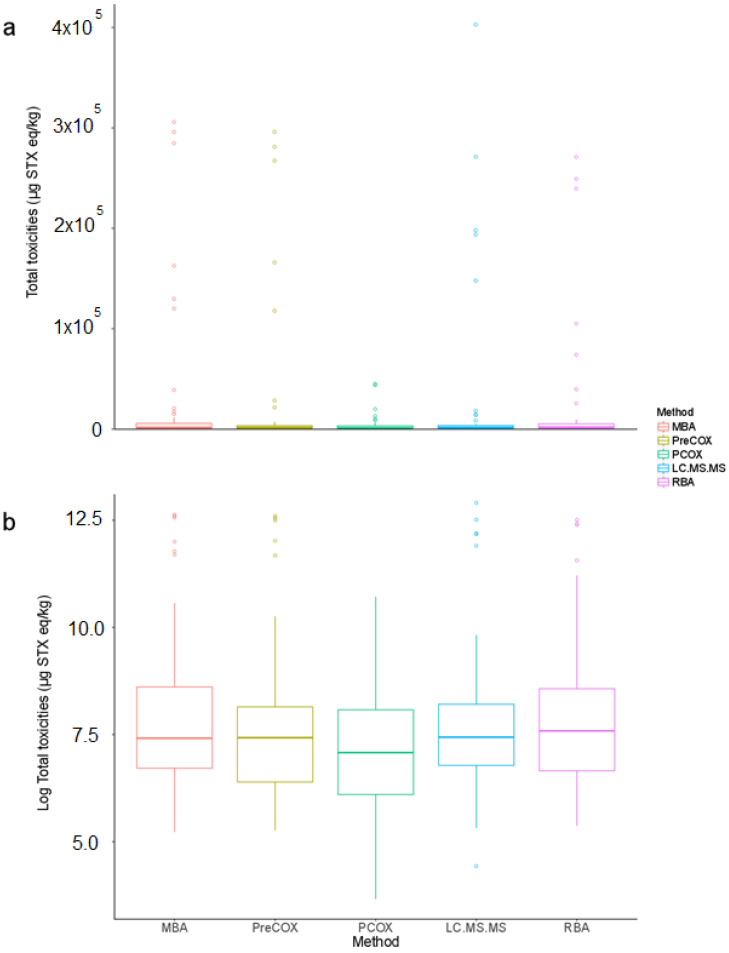
Distribution of test result values across five different test methods (MBA, PreCOX, PCOX, LCMSMS and RBA) with (**a**) raw data and (**b**) log-transformed data.

Across all quantitative data points, mean ratios between the total PST results obtained from each method in comparison with the PreCOX LC-FLD were 0.88 (PCOX), 1.34 (LC-MS/MS), 1.65 (RBA) and 3.91 (MBA). However, different numbers of analyses were performed for each method, so method comparison continued with the analysis of samples where either five or four quantitative method results were generated. Tables summarises the mean PSP toxicities for each shellfish type (mussels, clams, oysters, scallops, geoduck and miscellaneous) in each of the four LA regions, using five testing methods. In total, 57 shellfish samples contained total PST results for all of the five quantitative methods—PreCOX, PCOX, LC-MS/MS, RBA and MBA. The data were found to be skewed, but the log transformation successfully normalised the data and results, with similar median values and homogeneous variances being observed across the five test methods (Figure 3, Table S3).

**Figure 4:**
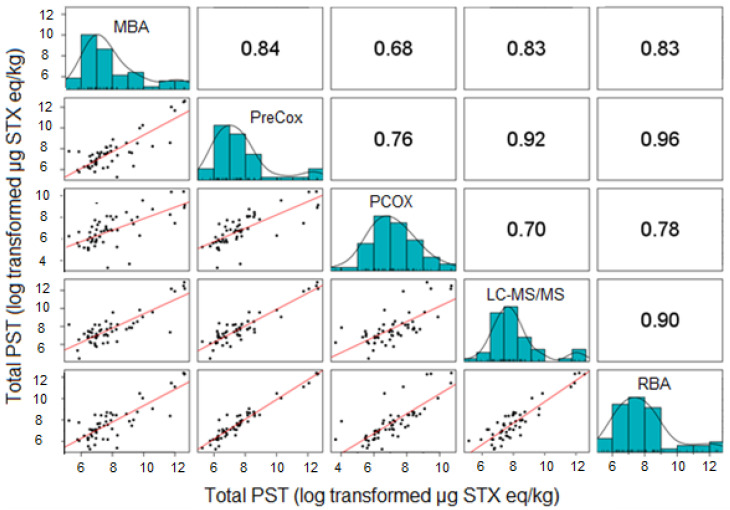
Correlation coefficients (upper half), scatter plots (lower half) and distributions (diagonal) for five different test methods (MBA, PreCOX, PCOX, LC-MS/MS and RBA) with samples taken within the same shellfish.

The scatter plots showed that the five methods were strongly correlated (Figure 4). The correlation coefficients were high and all of them were significant at the level of 5%. PCOX had, however, the lowest correlation coefficients (0.68 < r < 0.78), while all the other methods exhibited correlation coefficients higher than 0.80.

The repeated-measures ANOVA demonstrated that the different test methods explained a significant amount of the variability observed in the dataset (*p* = 0.004). Though the difference between the mean values obtained via each test method appeared small (Table S3), pairwise comparisons suggested that PCOX results were significantly different to the results of all the other methods, while PreCOX differed from the RBA (Table 2). The pairwise comparisons indicated no significant difference between the other methods at a level of 5%.

Overall, mean values determined for each species/country combination compared well between each method, with some exceptions. MBA data were found to be high in comparison to other method results for Argentinean scallops (n = 4), Chilean clams and mussels (n = 11), and Uruguayan samples (n = 7). Whilst the PCOX results compared generally well with those from other methods for the majority of samples, for Argentinean mussels, PCOX data were significantly lower than others. Whilst mussels from Argentina and Chile were associated most commonly with toxin profile 2, dominated by GTX1&4, other samples showing high relative MBA results were associated with toxin profile 1. High mean RBA values were obtained for Argentinean snails (n = 11), with the geoduck low RBA associated with just one sample.

#### 2.2.2. Comparison of Four Quantitative Methods

**Figure 5:**
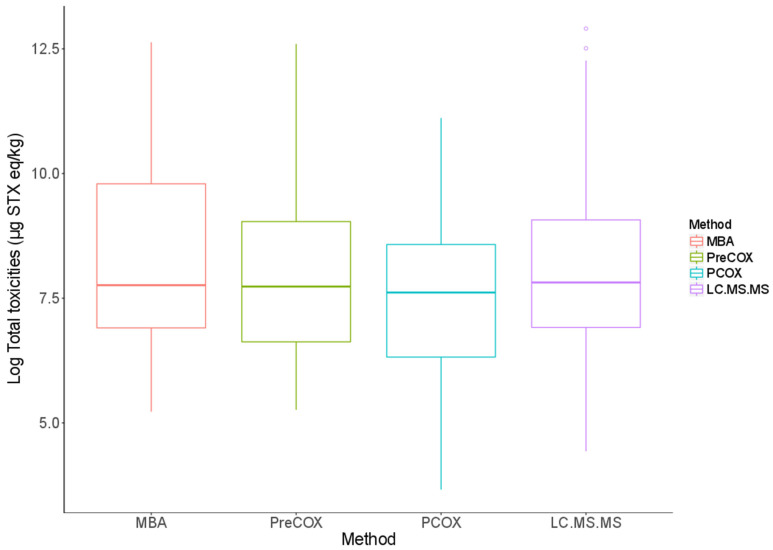
Distribution of test result values across four different test methods (MBA, PreCOX, PCOX and LC-MS/MS) with log-transformed data.

**Figure 6:**
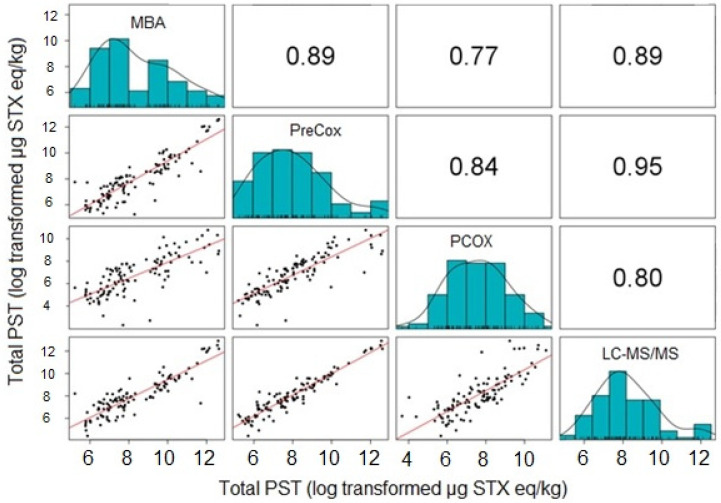
Correlation coefficient (upper half), scatter plot (lower half) and distribution (diagonal) for four different test methods (MBA, PreCOX, PCOX and LC-MS/MS) with samples taken within the same shellfish.

The previous analysis was repeated with the dataset excluding RBA, resulting in the comparative assessment of a larger total number of shellfish samples (n = 115). With approximately double the number of shellfish samples incorporated, differences were induced in the statistics of the method results compared to the previous dataset (Figure 5, Table S4). Table 3 summarises the mean PSP toxicity data obtained from each of the four methods. The scatter plots showed that the four methods were strongly correlated (Figure 6), which was confirmed with the high positive correlation coefficients. All of them were significant at the level of 5%.

The analysis of variance assessment demonstrated that the different test methods explained a significant amount of the variability observed in the dataset (*p* = 0.0005). Pairwise comparisons suggested that PreCOX results were not significantly different to the results of the LC-MS/MS method, while all the other pairwise comparison were significantly different at the level of 5% (Table 4). The conclusions from this analysis differed from the ones obtained with the dataset for the five methods. The larger number of samples is known to increase the power of tests used here. Therefore, a greater confidence was obtained with this second analysis. Table S4 summarises the mean total PST data generated using the four methods on the larger number of samples. High MBA data seen from the assessment of all five quantitative methods in Table 1 were confirmed using a larger number of samples with four testing methods in Argentinean scallops (n = 9), Chilean clams and mussels (n = 24) and Uruguayan clams (n = 3), although mussels from Uruguay showed a better comparison with a larger dataset (n = 7) (Table 3). Mean values again compared well with the other chemical detection methods with the exception of Argentinean mussels (n = 34).

#### 2.2.3. Comparison of Toxin Profiles between Methods

Figure 2a–c illustrate the mean toxin profiles determined for each of the three main profile clusters (1 to 3 inclusive) using all three chemical detection methods. Overall the profiles determined by LC-MS/MS are similar to those generated using both LC-FLD methods, with a clear dominance of STX in profile 1, GTX1&4 in profile 2 and with a similar spread of dcGTX2&3, C1&2 and dcSTX in samples associated with profile 3. The main difference observed relates to the additional incorporation of M-toxins into the LC-MS/MS, which are not detected by either LC-FLD method.

### 2.3. LFA vs. PreCOX LC-FLD

**Figure 7:**
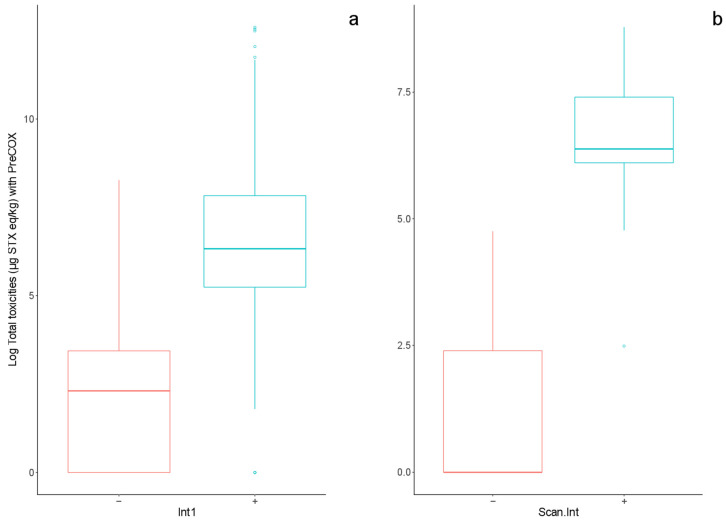
Comparison of log-transformed PreCOX test results associated with Scotia LFA negative (−) and positive (+) results with (**a**) visual interpretation “Int1” and (**b**) automated scan interpretation method “Scan.Int”.

A total of 250 shellfish extracts were analysed by Scotia LFA, with test cassettes interpreted visually by two analysts, resulting in 100% agreement between analysts. Two samples returned an invalid test strip result, with 199 positive results and 49 negative. Consequently, a dataset of 248 qualitative results were assessed in comparison with the reference PreCOX LC-FLD method. PreCOX values associated with both LFA positive and negative results were highly skewed. A log transformation normalised the data (Figure 7a). Welch’s two-sample *t*-test showed that PreCOX results were significantly higher in the group of positive LFA samples compared to the LC-FLD values determined in negative LFA samples at the level of 5%.

**Figure 8:**
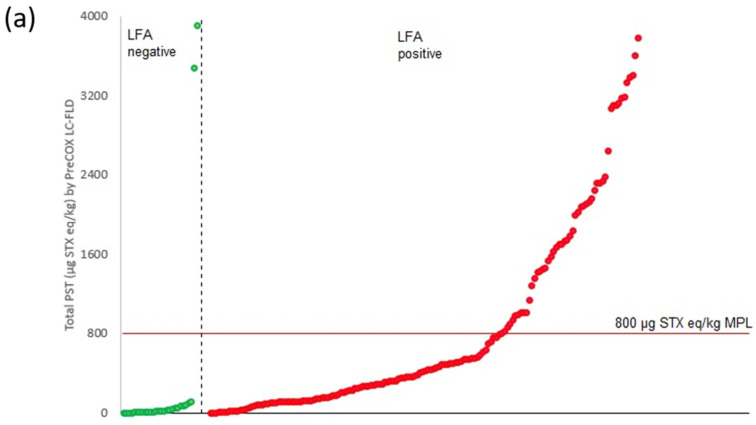

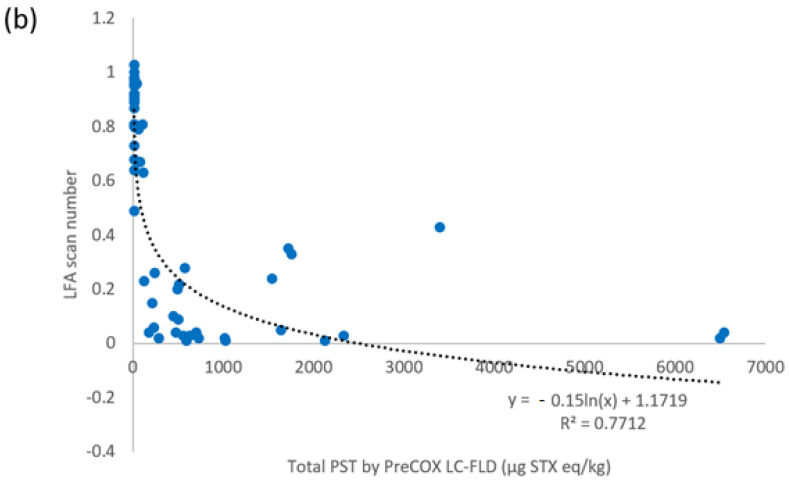
Total PST concentrations determined by PreCOX LC-FLD in comparison with (**a**) Scotia LFA qualitative results, highlighting MPL and (**b**) LFA scan number.

Figure 8 illustrates the qualitative LFA results obtained in comparison with the total PST concentrations determined by PreCOX LC-FLD. A total of 21 samples were found to contain no detectable levels of toxins by PreCOX, with 100% of these returning a negative LFA result. Further, 26 out of a total of 28 more negative LFA results exhibited total PST concentrations of <120 µg STX eq./kg by PreCOX LC-FLD, although the remaining two were highly toxic, with total PST of 3483 and 3914 µg STX eq./kg for a clam and gastropod sample, respectively. Out of the 199 positive LFA results following visual confirmation by two analysts, total PST concentrations determined by LC-FLD ranged from not detected to just below 400,000 µg STX eq./kg. A total of 106 LFA positive samples contained < 800 µg STX eq./kg toxicity by PreCOX, with a further 77 samples < 400 µg STX eq./kg which equates to the approximate, but profile-dependent limit of detection of the Scotia assay (Laycock et al., 2010). Consequently, out of 254 LFA tests conducted, 77 (30%) of these resulted in false-positive LFA results, with two false negatives (0.8%) (Table 5). The highest proportion of false-positive results were found in samples of Geoduck (66% of geoduck samples), followed by mussels and clams (both 20%), scallops (19%) and snails (9%).

A total of 56 LFA results were also assessed using the automated scanner, with LFA scan results confirming positive LFA results in 29 samples, with negative results in 27. Overall, there was good agreement between the visual and automated LFA results, with just four samples deemed positive by visual assessment, confirmed as negative by the automated approach (Table 5). PreCOX values associated with both LFA positive and negative results were again log transformed for normalising data (Figure 7b). However, the variance between the two groups remained heterogeneous, with much greater variability in PreCOX values observed in the negative LFA samples as compared to the positive group. Welch’s two-sample *t*-test, which does not require equal variances between groups, showed that PreCOX results were significantly higher in the LFA positive samples compared to the negative group at the level of 5%.

Scan numbers were produced for each of the automated scanner interpretation results from each of the LFA determinations. A regression was plotted between the scan numbers and PreCOX LC-FLD toxin concentrations, using 16 µg STX eq./kg for LC-FLD results showing no detectable toxins. Results showed the LFA scan numbers to decrease significantly with higher toxicity samples, with the regression showing a logarithmic correlation between the two parameters (y=−0.15lnx+1.1719) with a correlation coefficient r^2^ = 0.7712 (Figure 8b).

## 3. Discussion

### 3.1. PST Outbreaks in Latin America and Social Impacts

PST-producing harmful algal blooms are widely reported on an annual basis throughout Latin America, with regular occurrences of regiospecific toxigenic outbreaks of *A. catenella*, *G. catenatum* and *P. bahamense*. PST concentrations can periodically reach extraordinarily high levels in some regions resulting not only in extreme levels of risks to human health, but also significant impacts on animal health as exemplified by mass mortalities of marine mammals, and consequent socio-economic impacts [57,64,69]. The social impacts of this type of phenomenon are especially relevant in the coastal communities of southern Chile, whose traditions, gastronomy and subsistence are based on the ancestral relationship between the coastal communities and the sea. In this sense, precautionary closures make it impossible for coastal communities to use seafood as a source of food and small-scale marketing. Chile is the sixth-largest exporter of fish products in the world [106], an industry which is also a source of work and income for thousands of families, who are directly or indirectly related to the industry. For this reason, since 1995 the Shellfish National Sanitation Programme (PSMB), which is dependent on the National Fisheries and Aquaculture Services (Sernapesca), has maintained a surveillance system in the extraction areas and cultivation centres, whose products are mainly destined for export. In the year 2016, the worst PSP toxic event of all those recorded in terms of geographical extension and species affected occurred in the south of Chile. A total of 1700 people were unemployed due to HABs because of the inactivity of the processing plants and the ban on shellfish harvesting. Additionally, during this toxic episode, the highest mass mortality of invertebrates and vertebrates recorded was observed [70]. A particularly harmful case was observed on Cucao beach located on the Pacific Ocean coast of Chiloé island, where one of the most important commercial benthic fishing species was affected (macha: *Mesodesma donacium*) during the toxic outbreak, the main resource of the local economy of fishermen from the Huilliche ethnic group [70].

Consequently, in Chile and other LA countries, there has been an ongoing urgent need to ensure shellfish harvesting areas are monitored both routinely and effectively for the presence of potentially toxic microalgae and for biotoxins in shellfish flesh. Whilst the MBA has provided an effective monitoring tool for rapid assessment of PSP risks in shellfish for many decades, international laws are changing, resulting in the need for LA regions wishing to export shellfish to Europe and other regions to utilise non-animal bioassays for official control testing. For the internationally-validated methods to be used by LA countries, they must also be verified in house for applicability to the shellfish species of relevance, to generate performance characteristics and determine the size of measurement uncertainty. As part of these assessments, the comparison of multiple PSP detection methods would provide insights into the relative performance of each method, thereby benefitting the end users.

To date, various authors have reported the extent and distribution of total shellfish toxicity in each of the regions included in this study in a wide range of bivalve mollusc species [12,19,21,45,50,51,52,53,54,55,56,57,58,59,64,65,67,68,69], with more recent work describing PST analogues present in each of the four LA regions included in this study; Mexico, Uruguay, Chile and Argentina [44,57,61,64]. Analogues of saxitoxin reported are wide ranging including all the main gonyautoxins, carbamoyl and decarbamoyl congeners available as commercial reference materials, in addition to M toxins more recently reported [44,57,61,64]. Whilst the M toxins currently have no assigned toxicity equivalence factors (TEFs), the remaining analogues all contribute to total sample toxicity to varying extents through the continent, so any monitoring methods should be capable of detecting and quantifying the analogues of importance in each region.

### 3.2. Method Comparison

#### 3.2.1. MBA and RBA

Both the MBA and RBA should theoretically provide a direct and accurate determination of the total toxicity of any given shellfish sample. Whilst exhibiting low sensitivity, the MBA is generally thought to return similar total toxicity results when compared to chemical detection methods (e.g., [77,78,86,99]. There are well reported instances, however, where matrix effects cause significant underestimation of total toxicity by MBA, most notably in oysters containing naturally high concentrations of zinc [76,88,107,108], or where differences occur between methods related to different extraction steps and/or certain specific-source phytoplankton species such as *Gymnodinium catenatum* [103].

From the four LA regions studied here, statistical analysis demonstrated the MBA to correlate equally well with the RBA, PreCOX and LC-MS/MS methods, but with a lower correlation with PCOX (Figure 4). The correlation coefficient between MBA and PreCOX of 0.84 was higher than that reported previously in a multi-method assessment of PST concentrations in samples from Alaska [99] but similar to that from UK mussels [86]. Here, apparent differences in total PST following MBA in comparison with others were identified in certain species from some geographical regions, notably Chilean clams and mussels, Argentinean scallops and Uruguayan clams (Table 1 and Table 3). It is noted, however, that the MBA analyses were conducted several years prior to the other analyses, so there may be issues relating to either sample storage and/or transportation to other testing facilities. This does not explain, however, several instances of extreme outliers (defined here as results showing differences greater than an order of magnitude) for the MBA, specifically in three Argentinean mussels, one Argentinean scallop, two Chilean and one Uruguayan clam, all of which returned MBA results more than 10-fold higher than those determined using all other assays (Table S1).

The RBA, measuring toxicity through the direct measurement of the affinity of toxins to the sodium channel receptor, has been shown previously to compare generally well with the MBA for Chilean mussel and clam samples (r^2^ = 0.94, n = 41; [109]). Interestingly, an alternative functional electrophysiological assay for saxitoxins applied to a different set of 30 Chilean mussel and gastropod samples showed good correlation with the MBA (r^2^ = 0.90) but with the MBA consistently returning higher results [109]. During the RBA AOAC single-laboratory validation, which incorporated some shellfish samples from Chile, RBA toxicities were found to show a degree of overestimation in comparison to the MBA and PreCOX LC-FLD [91], as confirmed by more recent work based on mussels [110] and oysters [105], with the latter study confirming the RBA to be unaffected by high concentrations of zinc and other metals. In this study, RBA data further confirm its applicability to a wide range of shellfish species from wide-ranging geographical regions, with generally good correlation with the chemical detection methods, in particular with excellent correlations against both LC-MS/MS and PreCOX methods (Figure 4). Overall, these findings compare well with those from samples taken from Alaska, where the correlation between RBA and PreCOX was excellent (r^2^ = 0.95; [99]), although the RBA returned, on average, total toxicity results lower than those estimated from PreCOX LC-FLD analysis. Conversely, the mean RBA to PreCOX ratio in this study was 1.65, confirming the findings of [92,105,110].

#### 3.2.2. Instrumental Methods

As opposed to the MBA and RBA detection methods, all three instrumental chemical assays utilise external calibration standards for each individual toxin analogue to quantify toxin concentrations, subsequently estimating total saxitoxin equivalents through application of published TEFs. It is therefore important for any chemical assay utilised to be developed and validated to facilitate analytes which are likely to be present in any given region. In Latin American countries, it is therefore important for assays to be capable of incorporating analogues derived from all three genera of PST-producing microalgae, resulting in the requirement for including almost all known congeners, currently commercially available as certified reference materials. Table 6 summarises the PST analogues which are incorporated into each of the three chemical testing methods utilised in this study. All PST analogues incorporated into the LC-MS/MS method were detected in samples from this study, but to varying extents, resulting in three major profile clusters.

The LC-MS/MS assay is capable of analysing all analytes listed, although the importance of including M toxins and doSTX remains unclear given the absence of experimentally determined TEFs for these analogues. In this study, total PST concentrations correlated excellently with the current EU reference method, PreCOX LC-FLD with a Pearson correlation coefficient of 0.95 (Figure 6) and no significant differences between the method data (Table 2 and Table 4), with an overall mean LC-MS/MS to PreCOX ratio of 1.34. This confirms previous work which has also described an excellent agreement between the LC-MS/MS and PreCOX method [94].

The PreCOX LC-FLD is validated for all other remaining PSTs with the exception of dcGTX1&4, which to the authors knowledge has to date only been reported in certain specific clam species capable of enzymatic conversion to these congeners from GTX1&4 [111,112,113]. In this study, dcGTX1&4 was detected in only eleven samples, representing only 5% of the total samples exhibiting total PST toxin concentrations > 16 µg STX eq./kg, with the maximum contribution to toxicity found in one clam sample containing 10% dcGTX1&4. Overall, therefore, the absence of dcGTX1&4 monitoring for these samples would not greatly affect the accuracy of the final results. doSTX was quantified in 47% of samples, but at concentrations so low, the overall contribution to the total sample toxicity was negligible, with a mean and maximum total toxin proportion of 0.01% and 0.21%, respectively (Table S2). M toxins, detected in 80% of the samples currently have no TEF data formally assigned, but using current assumptions, total M toxin contributions to sample toxicity averaged 3.5%, with a maximum of 33%. Whilst there is consequently the potential for total PST concentrations to be underestimated using non-mass spectrometric instrumental methods such as PreCOX, further work is required to determine the correct TEFs for these analogues. Overall, however, whilst the PreCOX method showed statistically significant differences from results returned by other methods other than LC-MS/MS, the correlation coefficients associated with the pairwise comparisons were high, ranging from 0.76 to 0.96 (Figure 4).

Total toxin concentrations determined by PCOX LC-FLD also correlated well with results obtained by the other quantitative methods, although the correlation coefficients were lower than between the other methods, ranging from 0.68 to 0.78 (Figure 4) and with data deemed significantly different to those generated by all other methods (Table 2 and Table 4). Most notably, large differences were found for samples of Argentinean mussels, where PCOX total PST concentrations were lower than those calculated for all other methods (Table 1 and Table 3). The correlation coefficients determined here are similar to those reported from previous studies incorporating multiple shellfish matrices [83], although Alaskan researchers have shown a correlation coefficient (r^2^) of 0.96 between PCOX and MBA, with an average 200% toxicity result for PCOX in comparison to the bioassay [114]. Higher toxicity results from the PCOX in comparison to PreCOX LC-FLD methods have also been described [115], although the opposite has been reported by other authors when assessing PST concentrations in oysters [104]. The mean PCOX to PreCOX ratio in this study was 0.88 showing a close similarity in total PST concentrations overall, as determined by the two FLD methods. Whilst the method is fully validated for the majority of analogues, in addition to dcGTX1&4, notable exceptions include dcNEO and GTX6, both of which, along with other analogues can contribute significantly to total STX equivalents in shellfish associated with *G. catenatum* [27,116,117,118,119]. Consequently, it is important to assess local toxin profiles to help determine whether intended methods of analysis will successfully incorporate all present and relevant analogues. Samples from this study typically contained low relative proportions of dcNEO and GTX6, found in a total of 13% and 27% of samples, respectively (Table S2). Mean total toxin contributions from these two analogues were low (0.1% and 0.6%), reaching a maximum of 5.3% and 15% for dcNEO and GTX6, respectively. Consequently, there is the potential for underestimation of toxicity using PCOX for any samples containing high relative proportions of dcNEO and GTX6, given that the method used in this study was not extended to these two analogues. Overall, however, other than the findings with Argentinean mussels, the low PCOX to PreCOX ratio observed in samples in this study were not associated with any specific shellfish species, geographical region, or toxin profile cluster, so no clear patterns were found which may explain the observed differences between these methods.

#### 3.2.3. LFA

For LA regions wishing to export shellfish products to the US, the Scotia LFA can be used in certain situations as a screen for PST presence, hence there was a need to assess the comparative performance of this assay against validated regulatory methods. In this study, the Scotia LFA was assessed in direct comparison with the current EU reference method, PreCOX LC-FLD (n = 248). From a total of 49 LFA negative test results, all except two contained total PST concentrations < 15% of the 800 µg STX eq./kg MPL, although the two remaining negative LFA results related to samples containing PSP at more than 4-fold higher than the MPL. As such, whilst 96% of negative LFA results compared well with the PreCOX LC-FLD, the two false-negative LFA results were of concern given the high levels of toxins present. The two samples concerned were A48 and A54, a clam and snail sample from Argentina, respectively. Both samples were confirmed to contain high toxin concentrations by all other detection methods, evidencing the problem was not due to a PreCOX false positive. In addition, the LFA analysis was repeated on each sample, with the same findings. Toxin profiles for both the false-negative samples were assessed, with A48 containing 74% GTX1&4 together with a mix of C1&2 and GTX2&3, whereas toxicity in sample A54 resulted from 92% STX. Sample A54, containing almost exclusively STX would therefore not be expected to result in a false-negative LFA, given that the antibody was for STX. On the other hand, the high proportion of GTX1&4 in sample A48 may have been a factor. However, the refined LFA protocol was used, whereby GTX1&4 is transformed into an analogue with higher cross reactivity, resulting in a much lower chance of false-negative assay results [98]. Moreover, many other additional shellfish samples from this study contained very high proportions of GTX1&4 and did not result in false-negative LFA results.

All other samples containing PSTs as evidenced by PreCOX were found to result in a positive LFA result. A total of 30% of the LFAs conducted were, however, found to be false-positive results, with PreCOX showing toxins were either not detected or quantified at concentrations well below the MPL. Consequently, there is a risk that positive LFA results may be associated with sample batches that are safe for human consumption. These findings are similar to those reported previously by authors assessing the Scotia LFA in comparison to LC-FLD methods in samples from Alaska [99], the UK [98], the US [120,121] and Hong Kong [122].

Previous work also illustrated a logarithmic correlation between LFA scan number and total PST concentrations determined by PreCOX LC-FLD, with scan numbers < 0.3 equating to toxicities above the MPL [98]. Such a relationship was also evident overall in this study (Figure 8b). However, three samples, two scallops and one mussel, with total PST higher than twice the MPL, returned scan values > 0.3, making use of the semi-quantitative aspect of the scanner more risky. Overall, therefore, use of the Scotia LFA would be useful for reducing the number of quantitative confirmatory analyses required, particularly as a product batch test, but a fully validated quantitative assessment using an appropriate method would still be required for regulatory testing, noting the risks resulting from a high proportion of false-positive and the potential for occasional false-negative LFA results in samples with significant toxin loads.

### 3.3. Method Implementation

The EU is the main destination market for bivalve molluscs from South America [58], so the recent changes to EU shellfish safety legislation and the global move away from reliance on the MBA have placed pressures on LA regions to conduct official control testing using alternative methods. These pressures arise from the ethical aspects of utilising animal experimentation for food safety testing, and the legal aspects associated with exporting seafood to the EU and other regions where the MBA is no longer accepted as a regulatory monitoring tool [123,124]. In Europe, parts of North America and Australasia, alternative PSP detection methods have been used for both regulatory official control and commercial testing for many years. The PreCOX method is perhaps the most widely implemented currently, given its status as the EU reference method for PSP determination and its application nowadays in the UK and the majority of European member states [82] with developments to increase throughput and performance being adopted [125]. The PCOX method has been used for many years in Canada, and more recently selected by US States, given its acceptance by the Interstate Shellfish Sanitation Conference (ISSC) for shellfish growing area classification [126]. The RBA can also be used for regulatory control in mussels and for screening in clams and scallops [126]. More recently, the LC-MS/MS method has become the main official control method in New Zealand and Australia and the Scotia LFA is approved for use as a shellfish screening tool in the US [126]. Consequently, there is currently no single testing method available which is suitable for official control monitoring of bivalves destined for global export. Within LA, some countries have been working towards and achieving validation and accreditation of alternative testing methods for some time. In Mexico, where US exports are of high importance, PCOX (or alternative post-column protocols) has been utilised by some research laboratories since 2014 [64] but still this method still is not recognised in Mexican National Shellfish Sanitation Program. In contrast, the Scotia LFA was officially incorporated into this program in 2015 and used routinely for monitoring PSTs in shellfish. To date, however, its effectiveness has not been evaluated. Whilst the PreCOX method has been set up in monitoring laboratories in Chile, it has not been implemented for official controls. Similarly, both Uruguay and Argentina still utilise the MBA for official control testing, although work is underway to prepare for potential future implementation of chemical detection methods in Argentina.

Through the comparison of testing methods, this comparative study has helped to further demonstrate that across a wide range of shellfish species and geographical regions, the various alternative methods for PSP testing in bivalve molluscs are suitable for official control use. For those countries yet to move away from reliance on MBA, however, there is much work required in order to implement any such alternative methods. Replacement methods must be formally validated in each official control laboratory, with a particular focus on the species of commercial importance. In order to obtain accreditation to the relevant national or international quality standards, a robust series of controls and management processes also need to be developed and implemented [82]. Numerous challenges exist to LA regions, such as the four studied here, including the expense of instrumentation, often required in multiple establishments given the wide geographical expanse of many regions [60], the cost of consumables and toxin standards, instrument maintenance charges and the costs associated with training and maintaining the skills and experience of laboratory analysts.

## 4. Materials and Methods 

### 4.1. Samples

Shellfish samples were obtained from four countries within LA-Argentina, Chile, Mexico and Uruguay. The samples chosen for analysis included the major species of commercial importance to each country and were harvested between 1986 and 2012. These consisted of four species of mussels, oysters, five clam species, three scallop species, two marine snails and a selection of miscellaneous marine organisms (Table 7). A total of 263 samples were collected and analysed by the MBA before being transported to the UK for additional testing. An additional 86 samples were sent to the UK which were not subjected to MBA, making a total of 349 samples associated with this study. All samples were analysed by quantitative PreCOX LC-FLD, with other methods of analysis applied to selected samples as described below.

### 4.2. Reagents and Chemicals

Chemicals were LC-MS-reagent grade where possible, with sample preparation and solid-phase extraction reagents of HPLC grade (Rathburns, Walkerburn, UK). Mobile phases were prepared from LC-MS-grade solvents (Fisher Optima, ThermoFisher, Loughborough, UK). Certified reference materials for purified toxin standards of saxitoxin (STX), gonyautoxins 1–5 (GTX1-5), neosaxitoxin (NEO), decarbamoylsaxitoxin (dcSTX), N-sulfocarbamoyl gonyautoxin-2&3 (C1&2), decarbamoylneosaxitoxin (dcNEO) and decarbamoylgonyautoxin-2&3 (dcGTX2&3) were obtained from the Institute of Biotoxin Metrology, National Research Council Canada (NRCC, Halifax, Nova Scotia, Canada). Additional non-certified reference material standards of N-sulfocarbamoyl gonyautoxin-1&4 (C3&4), decarbamoylgonyautoxin-1&4 (dcGTX1&4) and gonyautoxin-6 (GTX6) were obtained from the NRCC. A reference standard for deoxydecarbamoylsaxitoxin (doSTX) was obtained from Cawthron Natural Compounds (CNC; Nelson, New Zealand).

For quantitation of PSTs by PreCOX LC-FLD, CRMs were diluted in ~4.5 mL water to form concentrated stock standard solutions and subsequently diluted in 0.1 mM acetic acid to form instrument calibration standards. Toxin standard mixes were prepared as recommended [73]. For PCOX LC-FLD analysis, concentrated stock solutions were prepared following AOAC 20011.02 [85], with primary GTX and STX toxin standards diluted in ~4.5 mL 0.3 mM HCl. C1&2 primary standards were diluted in ~4.5 mL pH5 water. Instrumental calibrants were prepared following further dilution in the same reagents. For HILIC-MS/MS analysis, 100 µL of each CRM was pipetted to form a mixed stock, containing C1&2, dcGTX2&3, GTX1-5, dcSTX, dcNEO, STX, NEO and doSTX. This solution was subsequently used to prepare calibration standards for HILIC-MS/MS by diluting the mixed stock solution into a diluent of carbon SPE-cleaned mussel extract, diluted as per samples to give a concentration of 80% acetonitrile (MeCN) with 0.25% acetic acid [95].

[^3^H] STX for the RBA was obtained from American Radiolabeled Chemicals (St. Louis, MO 63146, USA). Saxitoxin standard curves were prepared from 3 mM HCl dilutions of STX diHCl standard (NIST RM8642, US National Institute of Standards and Technology). The RBA buffer was 100 mM MOPS/100 mM choline chloride, pH 7.4. Rat brain membranes were prepared in bulk according to [92] and stored at −80 °C until use. Optiphase liquid scintillation cocktail (Perkin-Elmer Life Sciences, Downers Grove, IL USA) was used for scintillation counting.

No additional reagents were required for running the Scotia PSP testing method, other than those supplied in the testing kit.

### 4.3. Methods of Analysis

#### 4.3.1. Shellfish Extraction and MBA

Shellfish were shipped after sampling to the regional laboratories, where the molluscs were shucked and fleshy tissues extracted following AOAC Official Method 959.08 [74]. An amount of 100 g shellfish homogenates was mixed with 100 mL 0.1 M hydrochloric acid, with the pH adjusted to <4.0. The mixture was boiled gently for 5 min before cooling, re-adjusting the pH to 3.0–4.0 if required and centrifugation prior to analysis. The supernatant fluids were used for the assays. The MBAs were performed at each laboratory following individual laboratory standard operating procedures (SOPs), following the guidance of [74]. Sample toxicities were calculated from the median death times of the mice and expressed in terms of µg STX eq./kg shellfish flesh. The limit of detection (LOD) of the bioassays was between 300 and 350 µg STX eq./kg across all laboratories. The established guideline of 800 µg STX eq./kg was applied to determine whether harvesting areas were to be open or closed.

After completion of the MBA, remaining tissues and/or HCl extracts were stored frozen (<−15 °C) until required for further analysis. Subsamples of tissues and extracts were transported frozen under temperature-controlled conditions to Cefas. Samples were received from each laboratory after a maximum of five days transportation. Upon arrival in the UK, samples were checked and stored at −20 °C until required for analysis. Samples received as shellfish tissue homogenates were thawed and extracted in 1% acetic acid, following the double extraction procedure detailed by AOAC 2005.06 [73] and as standardised [86].

MBA data were generated in the relevant LA-based organisation soon after the shellfish samples had been obtained. After shipment of samples to Cefas, both PreCOX and PCOX LC-FLD analyses were conducted over a period of four months, in multiple batches, followed by LFA. Sample extracts were subsequently stored for a further four years before the LC-MS/MS was conducted at Cefas. At the same time, samples were sent to NOAA for RBA testing.

#### 4.3.2. PreCOX LC-FLD

Acetic acid and HCl extracts from the 349 shellfish samples were cleaned up using reverse-phase solid-phase extraction (SPE) with C18-bonded cartridges (C18-T SPE, Phenomenex, Manchester, UK). Eluants were adjusted to pH 6.5 ± 0.5 and diluted to 4.0 mL. C18-cleaned extracts were fractionated using a refined ion-exchange (COOH) SPE clean up [86]. C18-cleaned extracts of each sample were subjected to periodate oxidation [79] before qualitative LC-FLD analysis. All samples were subsequently quantified against 5 level calibration standards following peroxide oxidation of C18-cleaned extracts. Periodate oxidation of fractions F1-F3 was conducted prior to additional LC analysis for samples showing the potential presence of N-hydroxylated PSTs. Un-oxidised C18-cleaned extracts were also analysed with peak areas of any naturally-fluorescent chromatographic peaks subtracted from the toxin peak areas at the same retention time within the oxidised sample.

Liquid chromatography was conducted according to [86]. Mobile phases were those described by AOAC 2005.06 [73]. Agilent (Manchester, UK) 1200 series LC systems were used to deliver the mobile phase at a flow rate of 2 mL/min. Gemini C18 reversed-phase columns (150 mm × 4.6 mm, 5 µm; Phenomenex, Manchester, UK) were with a Gemini C18 guard pre-column (both held at 35 °C). The chromatographic gradient was as described by [86]. Agilent fluorescence detectors (1200 model FLD) were used for the detection of the PSP toxin oxidation products, with excitation and emission set to 340 and 395 nm, respectively. PST analogues incorporated into the PreCOX LC-FLD detection method are summarised in Table 6. Toxicity equivalence factors (TEFs) were taken from those published by EFSA [127].

#### 4.3.3. PCOX LC-FLD

For PCOX LC-FLD analysis, acidic extracts of 165 shellfish samples were deproteinated and analysed following AOAC 2011.02 [85]. The samples chosen were those found to contain total PST above 200 µg STX eq./kg. The post-column system consisted of an Agilent 1200 LC-FLD instrument with a quaternary LC pump, with the addition of two Agilent 1260 isocratic pumps and an external column oven. PST concentrations in sample extracts were quantified against five-point calibration standards with individual toxin concentrations and total saxitoxin equivalents determined. As per PreCOX LC-FLD, toxicity equivalence factors (TEFs) were taken from those published by EFSA [127]. Concentrations of individual toxins were calculated in units of STX di-HCl eq./kg and concentrations summed to estimate sample toxicities in terms of µg STX di-HCl eq./kg. PST analogues incorporated into the PCOX LC-FLD detection method are summarised in Table 6.

#### 4.3.4. UHPLC-HILIC-MS/MS

UHPLC-HILIC-MS/MS was conducted following the method described by [94] and validated by [95,96]. Crude sample extracts from 277 samples were cleaned up to remove salt-based interferences, using Supelclean ENVI-Carb 250 mg/3 mL SPE cartridges. De-salted extracts were collected in 20% MeCN + 0.25% acetic acid and further diluted in MeCN in polypropylene autosampler vials.

A Waters (Manchester, UK) Xevo TQ-S tandem quadrupole mass spectrometer (MS/MS) coupled to a Waters Acquity UPLC I-Class was used for analysis. Chromatography was conducted using a 1.7 µm, 2.1 × 150 mm Waters Acquity BEH Amide UPLC column with a Waters VanGuard BEH Amide guard cartridge. The columns were held at +60 °C, with samples held in the autosampler at +4 °C. The mobile phases, column treatment and analysis gradient were all as described by [94]. All Waters Xevo TQ-S parameters were as detailed by [95].

Quantitation was conducted against the response factors calculated for 14 PSTs present in the five-point calibration standards available as certified reference standards. The additional toxins (C3, C4, dcGTX1, dcGTX4 and GTX6) were quantified using experimentally determined relative response factors (RRF) [94]. Toxin concentrations were adjusted for recovery based on the recoveries determined in matrix spikes [95]. Toxicity equivalence factors (TEFs) for STX, NEO, dcSTX, dcNEO, dcGTX2&3, GTX1-6, C2 and C4 were taken from EFSA recommendations [127]. TEFs for other congeners C1, C3, dcGTX1&4, doSTX were taken from other published evidence [6,89,95]. Semi-quantitation of M-toxins was conducted using a RRF of 1.0 in comparison to the calibration response generated by the nearest structurally similar analogue with TEFs taken as 0.1 (M1, M3 and M5) and 0.3 (M2 and M4) as derived from EFSA TEF data for GTX5&6 and 11-hydroxy STX, respectively [95]. Individual toxin concentrations and total sample toxicity were calculated as above. A summary of the PST analogues incorporated into the HILIC-MS/MS detection method are summarised in Table 6.

#### 4.3.5. Lateral Flow Assay (LFA)

A total of 250 shellfish extracts were subjected to the PSP LFA [97] following the test kit instructions provided. Higher toxin concentrations added to the cassette result in the test line (T line) becoming fainter, with the intensity of the T line providing a visual qualitative indication of sample toxin content. The test sensitivity is approximately 250 µg STX eq./kg tissue in terms of the boundary between a positive and negative test result [97,100]. A refined version of the assay was utilised for the testing of samples in this study, as described and assessed previously by [95]. The modified protocol incorporated an additional hydrolysis step to convert GTX1&4 into NEO, thereby improving the detection of GTX1&4 toxins which normally have a very low cross reactivity [128]. A volume of 200 µL of acidic shellfish extracts were mixed with the hydrolysis reaction powder provided in the test kit and left standing at room temperature for 60 min. A volume of 100 µL of the solution was subsequently transferred into 400 µL of test kit buffer solution and mixed. A volume of 100 µL of the resulting solution was pipetted into the sample well on the test kit cartridge and left to develop for 30 min before interpreting the results. All 250 cassettes were interpreted visually, following the guidance provided on the test kit instructions, with assessments conducted by two independent analysts [95]. For a smaller number of samples, a scanner provided by Scotia Rapid Testing Ltd. was used for automatically interpreting qualitative results and providing a numerical result for each test (n = 56). Positive samples were those recording a scanner result <0.5.

#### 4.3.6. Receptor Binding Assay (RBA)

RBAs were performed on a subset of 117 samples. Analyses were performed as per AOAC OMA-2011-27 [93], with all assays conducted in a 96-well microtiter filter plate format with type GF/B glass fibre filters and 0.65 µm pore size Durapore support membranes (Millipore, Bedford, MA, USA) as described by [105]. Samples were run in triplicate, with each plate containing a seven-point STX calibration curve and a QC check sample in addition to samples. Assay components were added to each well in the following order: 35 µL assay buffer; 35 µL STX standard, QC check, or sample extract; 35 µL [^3^H] STX; and 105 µL membrane preparation. Assay plates were subsequently covered and incubated at 4 °C (1 h), filtered and rinsed twice with ice cold assay buffer while under vacuum. After removal of residual buffer, 50 µL Optiphase scintillation cocktail was added per well and the top of the plate sealed prior to incubation (30 min at room temperature) and counted using a Wallac Microbeta II microplate scintillation counter for 1 min/well.

Quality control criteria were applied as per Turner et al., 2018. Curve fitting was performed in Prism (Graph Pad Software, Inc., San Diego, CA, USA) using a sigmoidal dose–response curve with a variable slope. Sample quantification was carried out only on sample dilutions that fell on the linear part of the curve (B/B_o_ of 0.2–0.7), where B represents the bound [^3^H]STX (in counts per minute, CPM) in the sample and B_o_ represents the maximum bound [^3^H]STX (in CPM) in the absence of sample or cold STX standard. Where more than one dilution fell within 0.2–0.7 on the standard curve, all sample wells corresponding to these dilutions are used to calculate sample concentrations.

### 4.4. Data Assessment

#### 4.4.1. Toxin Results

Total PST concentrations generated by the two LC-FLD methods and LC-MS/MS were used to estimate sample toxicities, which could be compared with total PSP toxicities determined by MBA and RBA. Furthermore, the concentrations quantified by the three LC-based methods were used to assess toxin profiles throughout the dataset, with K-means clustering used to assess groups of similarly clustered profile patterns [129].

#### 4.4.2. Statistical Assessment

Statistical analysis was performed using R statistical software (R Core Team, 2016). The aim was first to compare the total toxin levels in shellfish measured with the five different quantitative methods—MBA, PreCOX, PCOX, LC-MS/MS and RBA. Measurements were assessed on samples for which toxins have been detected by all five methods, thereby precluding results where samples had not been analysed by one or more methodologies. Initially, the dataset was filtered to incorporate all values higher than 32 µg STX eq./kg only. Any sample for which a method did not detect toxins or for which a method detected a toxin at a level lower than this limit was removed from the dataset. Fewer samples were analysed with RBA than with the other methods, which lead to a small dataset of 55 samples. Therefore, a second assessment was conducted incorporating the four remaining methods with a larger filtered dataset of 112 samples. A third analysis aimed to evaluate the performance of Scotia LFA for discriminating positive and negative results. For this purpose, the results obtained by the LFA were compared with the quantitative results obtained by the PreCOX LC-FLD method.

For each dataset, relationships between the methods were compared graphically using box-and-whisker and scatter plots. The strength and significance of the relationships were assessed by Pearson’s correlation. Results obtained by each test method applied to each shellfish sample in the dataset were compared using a repeated-measures analysis of variance (ANOVA). Test results were assumed to be nested within shellfish samples in the model error structure. This analysis therefore focused on assessing the variability observed between test methods as opposed to between subjects. Where the test method was found to explain a significant amount of variability observed within the dataset at the level of 5%, pairwise *t*-tests using the ‘holm’ method of adjusting *p*-values for the effect of multiple comparisons were used to determine which tests produced results that significant differed from one another. The comparison of the results obtained by the LFA and PreCOX LC-FLD methods was based on a box-and-whisker plot and a Welsh two-sample *t*-test. The same approach was performed for qualitative LFA results obtained using both visual and automated interpretation.

## 5. Conclusions

Three hundred and forty-nine shellfish samples sourced from four regions within Latin America—Argentina, Mexico, Chile and Uruguay—were subjected to analysis for PSP toxins using six different validated detection methods, the MBA, RBA, PreCOX and PCOX LC-FLD, LC-MS/MS and the Scotia LFA. Total sample toxicities determined in a large number of different shellfish species harvested between 1986 and 2012 were found to vary enormously, ranging from no detectable toxin concentrations to total sample toxicities more than 500-fold higher than the regulatory MPL of 800 µg STX eq./kg, with wide-ranging toxin profiles also determined. Qualitatively, the methods generally compared well. Whilst datasets for some method comparisons were deemed significantly different, strong correlations were determined in the total PST data calculated from each of the quantitative methods. Notably, an excellent comparison was demonstrated between the current EU reference method, PreCOX LC-FLD, and the LC-MS/MS method, with conversely an apparent overestimation in PSP when using the MBA in comparison to other methods for some shellfish species. The rapid, portable LFA from Scotia was shown to be effective for detecting PSTs in the majority of PST-positive samples, although two false-negative test strip results in samples more than 4-foldhigher than the MPL and the high proportion of false positives determined showed that there are limitations in the applicability of the assay for official control testing. Overall, the data determined have shown the potential for numerous alternative methods for PSP testing in shellfish to be applied to samples from selected regions within LA. Any such replacement method needs to be formally validated and a range of quality management processes developed before it can be implemented into any routine monitoring programme within the region.

## Figures and Tables

**Table 1:** Summary of mean PSP toxicities (µg STX eq./kg) calculated for each shellfish type in the four LA regions based upon 57 samples for which all five quantitative methods were used.

Region	Shellfish	n	MBA	PreCOX	PCOX	LC-MS/MS	RBA
Argentina	Clams	9	1020	1174	959	2001	1544
	Mussels	11	107,912	103,352	10,899	111,426	86,292
	Scallops	4	10,427	1480	3277	5064	4147
	Snails	11	5622	6775	4377	3719	9283
Chile	Clams	3	40,717	2018	1933	1675	1829
	Mussels	8	3902	1756	1039	1547	2332
	Oysters	1	1070	870	523	859	1016
	Scallops	2	339	349	339	145	501
Mexico	Geoduck (w)	1	4120	1582	1277	3680	792
Uruguay	Clams	2	2315	356	259	785	380
	Mussels	5	5536	2395	1904	4743	2641

**Table 2:** *p*-values from pairwise comparisons using paired *t*-tests, with underlined results showing significant differences between method data.

	MBA	PreCOX	PCOX	LCMSMS
PreCOX	0.225			
PCOX	0.00035	0.006		
LCMSMS	0.766	0.884	0.004	
RBA	0.884	0.019	0.00003	0.884

**Table 3:** Summary of mean PSP toxicities (µg STX eq/kg) calculated for each shellfish type in the four LA regions based upon 115 samples for which four quantitative methods were used.

Region	Shellfish	n	MBA	PreCOX	PCOX	LC-MS/MS
Argentina	Clams	10	1020	1174	959	2001
	Mussels	34	62,878	53,633	11,240	59,801
	Scallops	9	13,508	4516	3912	7816
	Snails	23	5993	5591	4506	3586
Chile	Clams	5	24,692	1343	1204	1271
	Mussels	19	4981	2275	1886	2071
	Oysters	1	1070	870	523	859
	Scallops	3	336	414	439	143
Mexico	Geoduck (w)	1	4120	1582	1277	3680
Uruguay	Clams	3	1810	307	200	679
	Mussels	7	6667	5663	7284	9223

**Table 4:** Summary of *p*-values from pairwise comparisons using paired t tests, with underlined results showing significant differences between method data.

	MBA	PreCOX	PCOX	LC-MS/MS
PreCOX	6.50 × 10^−5^			
PCOX	7.20 × 10^−10^	1.20 × 10^−5^		
LC-MS/MS	0.0007	0.331	1.50 × 10^−5^	

**Table 5:** Summary of Scotia LFA performance in comparison with PreCOX LC-FLD reference method highlighting for each shellfish species the total number of tests, invalid tests, disagreements, positive results, negative results, false-negative and false-positive results.

	Mussel	Clam	Oyster	Geoduck	Snail	Scallop	Misc	Total
Total tests	84	41	6	73	32	16	2	254
Invalid tests	1	0	0	0	1	0	0	2
Disagreements ^a^	0	1	0	0	2	1	0	4
Total Pos	81	24	1	51	27	13	2	199
Total Neg	2	16	5	22	2	2	0	49
False Neg ^b^	0	1	0	0	1	0	0	2
False Pos ^c^	17	8	0	46	3	3	0	77

^a^ Disagreement between visual and automated test strip interpretation. ^b^ PreCOX LC-FLD > MPL, with negative LFA result. ^c^ PreCOX LC-FLD < 0.5 MPL with positive LFA result.

**Table 6:** Summary of PST analytes incorporated into each chemical testing method.

Analogue	PreCOX	PCOX	LC-MS/MS
STX	y	y	y
GTX2	y	y	y
GTX3	y	y	y
GTX1	y	y	y
GTX4	y	y	y
GTX5	y	y	y
GTX6	y	-	y
NEO	y	y	y
C1	y	y	y
C2	y	y	y
C3	y	y	y
C4	y	y	y
dcSTX	y	y	y
dcGTX2	y	y	y
dcGTX3	y	y	y
dcGTX1	-	-	y
dcGTX4	-	-	y
dcNEO	y	-	y
doSTX	-	-	y
M1	-	-	y
M2	-	-	y
M3	-	-	y
M4	-	-	y

y = analyte is incorporated into method; - = analyte is not incorporated into method.

**Table 7:** Summary of shellfish species analysed during the current study including shellfish type, species, common name and geographical source.

Samples	Species	Common Name	Source
Mussels	*Mytilus edulis*	Blue mussels	Uruguay, Argentina
	*Mytilus chilensis*	Chilean blue mussels	Chile
	*Aulacomya ater*	Ribbed mussels	Argentina
	*Brachidontes rodriguezii*	Scorched mussels	Argentina
Oysters	*Crassostrea gigas*	Pacific oysters	Chile
Clams	*Gari solida*	Gari clams	Chile
	Venus antiqua	Venus clams	Chile, Argentina
	*Donax hanleyanus*	Wedge clams	Uruguay, Argentina
	*Mesodesma mactroides*	Yellow clams	Argentina
	*Panopea globosa*	Gulf of California geoduck clam	Mexico
Scallops	*Argopecten purpuratus*	Scallops	Chile
	*Zygochlamys patagonica*	Patagonian scallops	Argentina
	*Aequipecten tehuelchus*	Tehuelche scallops	Argentina
Marine snails	*Adelomelon beckii*	Beck’s volute sea snail	Argentina
	*Zidona dufresnei*	Angular volute sea snail	Argentina
Squid	*Illex argentinus*	Mantle squid	Argentina
Miscellaneous	*Exact species unknown*	Limpets, shrimp heads	Argentina

## Supplementary Materials

The following are available online at https://www.mdpi.com/1660-3397/18/12/616/s1; Table S1. Summary of PST toxicities determined by MBA, PreCOX, PCOX, LC-MS/MS and RBA in Latin American shellfish samples, with Scotia LFA results summarised according to both visual and automated assessment including LFA scan number. (v) = viscera only; (s) = siphon only; (w) = whole tissues; nd = not detected; Pos = positive LFA results; Neg = negative LFA result. Scan value = numerical output from scanner (no units). Sample id prefix denote country of origin: A = Argentina, C = Chile, M = Mexico, and U = Uruguay. Blank cells infer no test conducted. Yellow shaded cells highlight extreme outliers (more than order of magnitude difference compared with mean of other results). Table S2. Summary of total PST (µg STX eq/kg) determined by LC-MS/MS, associated toxin profiles (in terms of proportion saxitoxin equivalents), cluster types and total number of toxin analogue detections for all Latin American shellfish samples exhibiting total PST > 16 µg STX eq/kg. Table S3. Summary of statistics (minimum, maximum, mean, median and variance) obtained from log-transformed PST toxicity data generated by the five quantitative testing methods (MBA, PreCOX, PCOX, LC-MS/MS and RBA). Table S4. Summary of statistics (minimum, maximum, mean, median and variance) obtained from log-transformed PST toxicity data generated by the four quantitative testing methods (MBA, PreCOX, PCOX and LC-MS/MS). Figure S1. Bar charts illustrating sample types associated with each toxin profile cluster, in relation to a) country of origin and b) shellfish species. Arg = Argentina, Mex = Mexico, and Uru = Uruguay. M = mussel, Cl = clam, Sc = scallop, G = gastropod, Ge = geoduck, O = oyster, and Misc = miscellaneous.

## References

[1] Hallegraef G.M., Hallegraef G.M., Anderson D.M., Cembella A.D. (2003). Harmful algal blooms: A global overview. Manual on Harmful Marine Microalgae.

[2] Llewellyn L., Negri A., Robertson A. (2006). Paralytic shellfish toxins in Tropical Oceans. Toxin Rev..

[3] Wiese M., D’Agostino P.M., Mihali T.K., Moffitt M.C., Neilan B.A. (2010). Neurotoxic alkaloids: Saxitoxin and its analogs. Mar. Drugs.

[4] FAO (Food and Agriculture Organisation of the United Nations) (2004). Marine Biotoxins, Paper 80.

[5] Etheridge S.M. (2010). Paralytic shellfish poisoning: Seafood safety and human health perspectives. Toxicon.

[6] Oshima Y. (1995). Post-column derivatisation liquid chromatography method for paralytic shellfish toxins. J. AOAC Int..

[7] Vale P. (2010). Metabolites of saxitoxin analogues in bivalves contaminated by *Gymnodinium catenatum*. Toxicon.

[8] Negri A.P., Bolch C.J.S., Geier S., Green D.H., Park T.-G., Blackburn S.I. (2007). Widespread presence of hydrophobic paralytic shellfish toxins in *Gymnodinium catenatum*. Harmful Algae.

[9] Lagos N. (2003). Paralytic shellfish poisoning phycotoxins: Occurrence in South America. Comments Toxicol..

[10] Montoya N.G., Akselman R., Carignan M.O., Carreto J.I. (2006). Pigment profile and toxin composition during a red tide of *Gymnodinium catenatum* Graham and *Myrionecta rubra* (Lohman) Jankowski in coastal waters off Mar del Plata, Argentina. Afr. J. Mar. Sci..

[11] Akselman R., Reguera B., Lion M. (2006). HAB-MAPS of toxic marine microalgae in coastal and shelf waters of South America. Proceedings of the 12th International Conference on Harmful Algae.

[12] Lewitus A., Horner R., Caron D., Garcia-Mendoza E., Hickey B., Hunter M., Huppert D., Kudela R., Langlois G., Largier J. (2012). Harmful algal blooms along the North American west coast region: History, trends, causes and impacts. Harmful Algae.

[13] Fabro E., Krock B., Torres A.I., Paparazzo F.E., Schloss I.R., Ferreyra G.A., Almandoz G.O. (2018). Toxigenic dinoflagellates and associated toxins in San Jorge Gulf, Argentina. Oceanography.

[14] Band-Schmidt C.J., Duran-Riveroll L.M., Bustillos-Guzmán J.J., Leyva-Valencia I., Lopez-Cortes D.J., López-Cortés D.J., Nuñez-Vázquez E.J., Hernández-Sandoval F.E., Ramirez-Rodriguez D.V. (2019). Paralytic toxin producing dinoflagellates in Latin America: Ecology and physiology. Front. Mar. Sci..

[15] Balech E. (1964). El plancton de Mar del Plata durante el periodo 1961–1962. Bol. Inst. Biol. Mar..

[16] Akselman R., Carreto J.I., Montoya N.G., Reguera B., Blanco J., Fernández M.L., Wyatt T. (1998). Gymnodinium catenatum and autumn toxicity in northern shelf waters of Argentina. Harmful Algae.

[17] Andrinolo D., Santinelli N., Otano S., Sastre V., Lagos N. (1999). Paralytic shellfish toxins in mussel and *Alexandrium tamarense* at Valdes Peninsula, Chubut, Patagonia Argentina: Kinetics of a natural depuration. J. Shellfish Res..

[18] Santinelli N., Gaille G., Lettieri A., Intergovernmental Oceanographic Commission (IOC) of UNESCO (1994). Harmful algae and PSP toxicity along north Patagonia coast. Harmful Algae News.

[19] Carreto J.I., Montoya N., Colleoni A.D.C., Akselman R., Reguera B., Blanco J., Fernández M.L., Wyatt T. (1998). Alexandrium tamarense blooms and shellfish toxicity in the Argentine Sea: A retrospective view. Harmful Algae.

[20] Gayoso A.M. (2001). Observations on *Alexandrium tamarense* (Lebour) Balech and other dinoflagellate populations in Golfo Nuevo, Patagonia (Argentina). J. Plankton Res..

[21] Benavides H., Prado L., Diaz S., Carreto J.I., Lassus P., Arzul G., Erard E., Gentien P., Marcaillou C. (1995). An exceptional bloom of Alexandrium catenella in the Beagle Channel, Argentina. Harmful Marine Algal Blooms, Proceedings of the Sixth International Conference on Toxic Marine Phytoplankton, Nantes, France, 18–22 October 1993.

[22] Almandoz G.O., Harnando M.P., Ferreyra G.A., Schloss I.R., Ferrario M.E. (2011). Seaonsal phytoplankton dynamics in extreme southern South America (Beagle Channel, Argentina). J. Sea Res..

[23] Almandoz G.O., Cefarelli A.O., Diodato S., Montoya N.G., Benavides H.R., Carigan M., Hernando M., Fabro E., Metfies K., Lundholm N. (2019). Harmful phytoplankton in the Beagle Channel (South America) as a potential threat to aquaculture activities. Mar. Pollut. Bull..

[24] Brazeiro A., Mendez S.M., Ferrari G. (1997). First toxic bloom of *Alexandrium tamarense* in Uruguay: Associated environmental factors. Rev. Atl..

[25] Mendez S., Ferrari G. (2003). Floraciones toxicas de *Gymnodinium catenatum* en aguas uruguayas. Publ. Com. Tec. Mix. Frente Marit..

[26] Mendez S.M., Medina D., Steidinger K.A., Landsberg J.H., Tomas C.R., Vargo G.A. (2004). Twenty-three years of red tide monitoring at fixed stations along the coast of Uruguay. Harmful Algae 2002.

[27] Garate-Lizarraga I., Bustillos-Guzman J.J., Morquecho L., Band-Schmidt C.J., Alonso-Rodriguez R., Erler K., Luckas B., Reyes-Salinas A., Gongora-Gonzalez D.T. (2005). Comparative paralytic shellfish toxin profiles in the strains of *Gymnodinium catenatum* Graham from the Gulf of California, Mexico. Mar. Poll. Bull..

[28] Band-Schmidt C.J., Bustillos-Guzmán J.J., López-Cortés D.J., Gárate-Lizárraga L., Nuñez-Vázquez E.J., Hernández-Sandoval F.E. (2010). Ecological and physiological studies of *Gymnodinium catenatum* in the Mexican Pacific: A review. Mar. Drugs.

[29] Ochoa J.L., Sánchez-Paz A., Cruz-Villacorta A., Nuñez-Vázquez E., Sierra Beltrán A. (1997). Toxic events in the northwest Pacific coastline of Mexico during 1992–1995: Origin and impacts. Hydrobiologica.

[30] Bustillos-Guzmán J.J., Leyva-Valencia I., Hernández-Sandoval F.E., Band-Schmidt C.J., López-Cortés D.J., Núñez-Vázquez E.J., García-Mendoza E., Quijano-Scheggia S.I., Olivos-Ortíz A., Núñez-Vazquez E.J. (2016). Ficotoxinas en aguas del Golfo de California: Una revision. Florecimientos Algales Nocivos en México.

[31] Santiago-Morales S.I., García-Mendoza E., Quijano-Scheggia S.I., Olivos-Ortíz A., Núñez-Vazquez E.J. (2016). Florecimientos algales nocivos en la costa de Oaxaca. Florecimientos Algales Nocivos en México.

[32] Morquecho L. (2019). *Pyrodinium bahamense* one of the most significant harmful dinoflagellate in Mexico. Front. Mar. Sci..

[33] Hernandez-Becerrill D.U., Lau W.L.S., Hil K.S., Leaw C.P., Varona-Cordero F., Lim P.T. (2018). Abundance and distribution of the potentially toxic thecate dinoflagellate *Alexandrium tamiyavanichii* (Dinophyceae) in the central Mexican Pacific, using the quantitative PCR method. Front. Mar. Sci..

[34] Altamirano R.C., Sierra-Beltrán A.P. (2008). Biotoxins from freshwater and marine harmful algal blooms occurring in Mexico. Toxin Rev..

[35] Okolodkov Y.B., Garate-Lizárraga I. (2006). An annotated checklist of dinoflagellates (*Dinophyceae*) from the Mexican Pacific. Acta Bot. Mex..

[36] Hernández-Becerril D.U., Alonso-Rodiguez R., Alvarez-Góngora C., Barón-Campis S.A., Ceballos-Corona G., Herrera-Silveira J., Meave del Castillo M.E., Juárez-Ruiz N., Merino-Virgilio F., Morales-Blake A. (2007). Toxic and harmful marine phytoplankton and microalgae (HABs) in Mexican Coasts. J. Environ. Sci. Health Part A.

[37] Guzman L., Vivanco X., Vidal G., Pizarro G., Hernández C., Tocornal M.A., Pagou K.A., Hallegraeff G.M. (2013). Spatial and temporal variability of *Alexandrium catenella* and PSP in southern Chile (438–558S) (May 2006–July 2010). Proceedings of the 14th International Conference on Harmful Algae, Hersonissos, Crete, Greece, 1–5 November 2010.

[38] Hernández C., Díaz P.A., Molinet C., Seguel M. (2016). Exceptional climate anomalies and northwards expansion of Paralytic Shellfish Poisoning outbreaks in Southern Chile. Harmful Algae News.

[39] Tufts N.R., Taylor D.L., Seliger H.H. (1979). Molluscan transvectors of paralytic shellfish poisoning. Toxic Dinoflagellate Blooms.

[40] Shumway S.E. (1995). Phycotoxin-related shellfish poisoning: Bivalve molluscs are not the only vectors. Rev. Fish. Sci..

[41] Lasta M., Ciocco N., Bremec C., Roux A. (1998). Moluscos bivalvos y gasterópodos. El Mar Argentino y sus Recursos Pesqueros.

[42] Deeds J.R., Landsberg J.H., Etheridge S.M., Pitcher G.C., Longan S.W. (2008). Non-traditional vectors for paralytic shellfish poisoning. Mar. Drugs.

[43] Turner A.D., Tarnovius S., Goya A.B. (2014). Paralytic shellfish toxins in the marine gastropod *Zidona Dufresnei* and *Adelomelon Beckii* from Argentina: Toxicity and toxin profiles. J. Shellfish Res..

[44] Goya A.B., Tarnovius S., Hatfield R.G., Coates L., Lewis A.M., Turner A.D. (2020). Paralytic shellfish poisoning toxicity and associated profiles in bivalve mollusc shellfish from Argentina. Harmful Algae.

[45] Elbusto C.A., Ballabene A.C., Campero C.M., Ramírez E.E., Villanueva C.R. (1981). Toxina paralizante de los moluscos del Mar Argentino. Acta Bioquímica Latinoam..

[46] Carreto J.I., Lasta M.I., Negri R.M., Benavides H.R. (1981). Los fenómenos de marea y toxicidad de moluscos bivalvos en el Mar Argentino. Contr. Inst. Nac. Investig. Des. Pesq..

[47] Carreto J.I., Elbusto C., Sancho H., Carignan M., Yasumoto T., Oshima Y. (1996). Comparative studies on Paralytic Shellfish Toxin profiles of marine snails, mussels and an *Alexandrium tamarense* isolate from the Mar Del Plata coast (Argentina). Rev. Investig. Des. Pesq..

[48] Carreto J.I., Montoya N.G., Akselman R., Negri R.M., Carignan M.O., Cucchi Colleoni D.A., Steidinger K.A., Landsberg J.H., Tomas C.R., Vargo G.A. (2002). Differences in the PSP toxin profiles of *Mytilus Edulis* during spring and autumn blooms of *Alexandrium tamarense* off Mar Del Plata coast, Argentina. Harmful Algae 2002.

[49] Goya A.B., Maldonado S., Sauve G. (2014). Evolution of PSP toxicity in shellfish from the Beagle Channel (Tierra del Fuego, Argentina): An overview. Molluscan Shellfish Safety.

[50] Guzmán L., Campodónico I., Antunovic M. (1975). Estudios sobre un florecimiento tóxico causado por *Gonyaulax catenella* en Magallanes. Distribución y niveles de toxicidad de veneno paralítico de los mariscos (Noviembre de 1972–Noviembre de 1973). Ans. Inst. Pat. Punta Arenas.

[51] Carreto J.I., Benavides H.R., Negri R.H., Glorioso P.D. (1986). Toxic red tide in the Argentine Sea. Phytoplankton distribution and survival of the toxic dinoflagellate *Gonyaulax excavate* in frontal area. J. Plankton Res..

[52] Esteves J.L., Santinelli N., Sastre V., Diaz R., Rivas O. (1992). A toxic dinoflagellate bloom and PSP production associated with upwelling in Golfo Nuevo, Patagonia Argentina. Hydrobiologica.

[53] Ciocco N. (1995). La marisquería mediante buceo en el Golfo San José (Chubut, Argentina). Informe Técnico del Área de Pesca del Plan de Manejo Integrado de la Zona Costera Patagónica (GEF-PNUD, Patagonia, Argentina).

[54] Carreto J.I., Akselman R., Benavides H., Montoya N.G., Negri R., Sar E.A., Ferrario M.E., Reguera B. (2002). El proyecto “Marea Roja” del Instituto Nacional de Investigación y Desarrollo Pesquero. Floraciones Algales Nocivas en el Cono Sur Americano.

[55] Guzmán L., Pacheco H., Pizarro G., Alarcón C., Sar E.A., Ferrario M.E., Reguera B. (2002). Alexandrium catenella y veneno paralizante de los mariscos en Chile. Floraciones Algales Nocivas en el Cono Sur Americano.

[56] Persich G.R., Kulisb D.M., Lilly E.L., Anderson D.M., Garcia V.M.T. (2006). Probably origin and toxin profile of *Alexandrium tamarense* (Lebour) Balech from southern Brazil. Harmful Algae.

[57] Montoya N.G., Carignan M.O., Carreto J.I., Hoffmeyer M.S., Sabatini M., Brandini F.P., Calliari D.L., Santinelli N.H. (2018). Alexandrium tamarense/catenella blooms in the Southwestern Atlantic: Paralytic shellfish toxin production and its trophic transference. Plankton Ecology of the Southwestern Atlantic.

[58] Villalobos L.G., Santinelli N.H., Sastre A.V., Marino G., Almandoz G.O. (2019). Spatiotemporal distribution of paralytic shellfish poisoning (PSP) toxins in shellfish from Argentine Patagonian coast. Heliyon.

[59] Medina D., Inocente G., López C., Smayda T.J., Shimizu Y. (1993). PSP in bivalve molluscs along the Uruguayan coast. Toxic Phytoplankton Blooms in the Sea, Proceedings of the Fifth International Conference on Toxic Marine Phytoplankton, Newport, RI, USA, 28 October–1 November 1991.

[60] Medina D., Goya A.B., Rozas C., Sauvé G. (2014). Molluscan shellfish safety in South America. Molluscan Shellfish Safety.

[61] Turner A.D., Tarnovius S., Medina D., Salhi M. (2015). Use of a liquid chromatographic method for assessment of paralytic shellfish poisoning toxin profiles in mussels and clams from Uruguay. J. Shellfish Res..

[62] Saldate-Castañeda O., Vázquez-Castellanos J.L., Galván J., Sánchez-Anguiano A., Nazar A. (1991). Intoxicaciones por toxina paralizante de molusco en Oaxaca. Salud Publica Mex..

[63] Gárate-Lizárraga I., Bustillos-Guzmán J.J., López-Cortex D.J., Hernández-Sandoval F., Erler K., Luckas B. (2006). Paralytic shellfish toxin profiles in net phytoplankton samples from Bahía Concepción, Gulf of California, Mexico. Mar. Poll. Bull..

[64] Medina-Elizalde J., Garcia-Mendoza E., Turner A.D., Sanchez-Bravo Y.A., Murillo-Martinez R. (2018). Transformation and depuration of paralytic shellfish toxins in the geoduck clam *Panopea globosa* from the Northern Gulf of California. Front. Mar. Sci..

[65] García C., Bravo C., Lagos M., Lagos N. (2004). Paralytic shellfish poisoning: Post-mortem analysis of tissue and body fluid samples from human victims in the Patagonia fjords. Toxicon.

[66] Díaz P.A., Álvarez G., Varela D., Pérez-Santos I., Díaz M., Molinet C., Seguel M., Aguilera-Belmonte A., Guzmán L., Uribe E. (2019). Impacts of harmful algal blooms on the aquaculture industry: Chile as a case study. Perspect. Phycol..

[67] Molinet C., Niklitschek E., Seguel M., Diaz P. (2010). Trends of natural accumulation and detoxification of paralytic shellfish poison in two bivalves from the northwest Patagonian inland sea. Rev. Biol. Mar. Oceanogr..

[68] Alvarez G., Diaz P.A., Godoy M., Araya M., Ganuza I., Pino R., Alvarez F., Rengel J., Hernandez C., Uribe E. (2019). Paralytic shellfish toxins in surf clams *Mesodesma donacium* during a large bloom of *Alexandrium catenella* dinoflagellates associated to an intense shellfish mass mortality. Toxins.

[69] Terrazas J.O., Contreras H.R., Garcia C. (2017). Prevalence, variability and bioconcentration of saxitoxin-group in different marine species present in the food chain. Toxins.

[70] Strub P.T., James C., Montecino V., Rutlant J.A., Blanco J.L. (2019). Ocean circulation along the southern Chile transition region (38°–46°S): Mean, seasonal and interannual variability, with a focus on 2014–2016. Prog. Oceanogr..

[71] Trainer V.L., Moore S.K., Hallegraef G., Kudela R.M., Clement A., Mardones J.I., Cochlan W.P. (2020). Pelagic harmful algal blooms and climate change: Lessons from nature’s experiments with extremes. Harmful Algae.

[72] Anon (2004). Regulation (EC) No 854/2004 of the European Parliament and of the Council of 29th April 2004 laying down specific rules for the organisation of official controls on products of animal origin intended for human consumption. Off. J. Eur. Union.

[73] Anon (2005). AOAC Official method 2005.06 Quantitative Determination of Paralytic Shellfish Poisoning Toxins in Shellfish Using Pre-Chromatographic Oxidation and Liquid Chromatography with Fluorescence Detection.

[74] Anon (2005). AOAC Official method 959.08. Paralytic shellfish poison. Biological method. Final action. AOAC Official Methods for Analysis.

[75] Anon, Committee on Toxicity of Chemicals in Food, Consumer Products and the Environment Statement on Risk Assessment and Monitoring of Paralytic Shellfish Poisoning (PSP) Toxins in Support of Human Health. COT Statement 2006/08. July 2006. http://www.food.gov.uk/science/ouradvisors/toxicity/statements/cotstatements2006/cotstatementpsp200608.

[76] Turner A.D., Dhanji-Rapkova M., Algoet M., Suarez-Isla B.A., Cordova M., Caceres C., van de Riet J., Murphy C.J., Case M., Lees D.N. (2012). Investigations into matrix components affecting the performance of the official bioassay reference method for quantitation of paralytic shellfish poisoning toxins in oysters. Toxicon.

[77] Lawrence J.F., Me’nard C., Cleroux C. (1995). Evaluation of prechromatographic oxidation for liquid chromatographic determination of paralytic shellfish poisons in shellfish. J. AOAC Int..

[78] Lawrence J.F., Niedzwiadek B., Menard C. (2004). Quantitative determination of paralytic shellfish poisoning toxins in shellfish using prechromatographic oxidation and liquid chromatography with fluorescence detection: Interlaboratory study. J. AOAC Int..

[79] Lawrence J.F., Niedzwiadek B., Menard C. (2005). Quantitative determination of paralytic shellfish poisoning toxins in shellfish using prechromatographic oxidation and liquid chromatography with fluorescence detection: Collaborative study. J. AOAC Int..

[80] Anon (2006). Commission Regulation (EC) No 1664/2006 of 6th Nov. 2006 amending Regulation (EC) No 2074/2005 as regards implementing measures for certain products of animal origin intended for human consumption and repealing certain implementing measures. Off. J. Eur. Union.

[81] Anon (2017). Commission regulation (EU) No 2017/1980 of 31 October 2017 ammending Annex III to Regulation (EV) No 2074/2005 as regards paralytic shellfish poison (PSP) detection method. Off. J. Eur. Union.

[82] Turner A.D., Hatfield R.G., Maskrey B.H., Algoet M., Lawrence J.F. (2019). Evaluation of the new European Union reference method for paralytic shellfish toxins in shellfish: A review of twelve years regulatory monitoring using pre-column oxidation LC-FLD. Trends Anal. Chem..

[83] Van de Riet J.M., Gibbs R.S., Chou F.W., Muggah P.M., Rourke W.A., Burns G., Thomas K., Quilliam M.A. (2009). Liquid chromatographic post-column oxidation method for analysis of paralytic shellfish toxins in mussels, clams, scallops, and oysters: Single-laboratory validation. J. AOAC Int..

[84] Van de Riet J.M., Gibbs R.S., Muggah P.M., Rourke W.A., MacNeil J.D., Quilliam M.A. (2011). Liquid chromatographic post-column oxidation (PCOX) method for the determination of paralytic shellfish toxins in mussels, clams, oysters and scallops: Collaborative study. J. AOAC Int..

[85] Anon (2011). AOAC official method 2011.02 determination of paralytic shellfish poisoning toxins in mussels, clams, oysters and scallops. Post-Column Oxidation Method (PCOX). First Action 2011.

[86] Turner A.D., Norton D.M., Hatfield R.G., Morris S., Reese A.R., Algoet M., Lees D. (2009). N Single laboratory validation of the AOAC HPLC method (2005.06) for mussels: Refinement and extension of the method to additional toxins. J. AOAC Int..

[87] Turner A.D., Norton D.M., Hatfield R.G., Rapkova-Dhanji M., Algoet M., Lees D.N. (2010). Single laboratory validation of a refined AOAC LC method for oysters, cockles and clams in UK shellfish. J. AOAC Int..

[88] Turner A.D., Hatfield R.G., Rapkova M., Higman W., Algoet M., Suarez-Isla B.A., Cordova M., Caceres C., van de Riet J., Gibbs R. (2011). Comparison of AOAC 2005.06 LC official method with other methodologies for the quantitation of paralytic shellfish poisoning toxins in UK shellfish species. Anal. Bioanal. Chem..

[89] Harwood D.T., Selwood A.I., van Ginkel R., Waugh C., McNabb P.S., Munday R., Hay B., Thomas K., Quilliam M.A., Malhi N. (2014). Paralytic shellfish toxins, including deoxydecarbamoyl-STX, in wild-caught Tasmanian abalone (*Halitotis rubra*). Toxicon.

[90] NSSP (2013). National Shellfish Sanitation Program (NSSP) Guide for the Control of Molluscan Shellfish. 2013 Revision. http://www.fda.gov/downloads/Food/GuidanceRegulation/FederalStateFoodPrograms/UCM415522.pdf.

[91] Van Dolah F.M., Keighfield T.A., Doucette G.J., Bean L., Niedzwiadek B., Rawn D.F.K. (2009). Single laboratory validation of the microplate receptor binding assay for paralytic shellfish toxins in shellfish. J. AOAC Int..

[92] Van Dolah F.M., Fire S.E., Keighfield T.A., Mikulski C.M., Doucette G.J. (2012). Determination of paralytic shellfish toxins in shellfish by receptor binding assay: Collaborative study. J. AOAC Int..

[93] Anon (2011). AOAC Official Method 2011.27. Paralytic Shellfish Toxins (PSTs) in Shellfish, Receptor Binding Assay.

[94] Boundy M.J., Selwood A.I., Harwood D.T., McNabb P.S., Turner A.D. (2015). Development of a sensitive and selective liquid chromatography-mass spectrometry method for high throughput analysis of paralytic shellfish toxins using graphitised carbon solid phase extraction. J. Chromatogr. A.

[95] Turner A.D., McNabb P.S., Harwood D.T., Selwood A.I., Boundy M.J. (2015). Single laboratory validation of a multi-toxin UPLC-HILIC-MS/MS method for quantitation of paralytic shellfish toxins in bivalve shellfish. J. AOAC Int..

[96] Turner A.D., Dhanji-Rapkova M., Fong S.Y.T., Hungerford J., McNabb P.S., Boundy M.J., Harwood D.T. (2020). Ultrahigh-performance hydrophilic interaction liquid chromatography with tandem mass spectrometry method for the determination of paralytic shellfish toxins and tetrodotoxin in mussels, oysters, clams, cockles and scallops: Collaborative study. J. AOAC Int..

[97] Laycock M.V., Donovan M.A., Easy D.J. (2010). Sensitivity of lateral flow tests to mixtures of saxitoxins and applications to shellfish and phytoplankton monitoring. Toxicon.

[98] Turner A.D., Tarnovius S., Johnson S., Higman W.A., Algoet M. (2015). Testing and application of a refined rapid detection method for paralytic shellfish poisoning toxins in UK shellfish. Toxicon.

[99] Costa P.R., Baugh K.A., Wright B., RaLonde R., Nance S.L., Tatarenkova N., Etheridge S.M., Lefebvre K.A. (2009). Comparative determination of paralytic shellfish toxins (PSTs) using five different toxin detection methods in shellfish species collected in the Aleutian Islands, Alaska. Toxicon.

[100] Humpage A.R., Magalhaes V.F., Froscio S.M. (2010). Comparison of analytical tools and biological assays for detection of paralytic shellfish poisoning toxins. Anal. Bioanal. Chem..

[101] Rodriguez P., Alfonso A., Botana A.M., Vieytes M.R., Botana L.M. (2010). Comparative analysis of pre- and post-column oxidation methods for detection of paralytic shellfish toxins. Toxicon.

[102] DeGrasse S.L., Van de Riet J., Hatfield R., Turner A.D. (2011). Pre- versus post-column oxidation liquid chromatography fluorescence detection of paralytic shellfish toxins. Toxicon.

[103] Ben-Gigirey B., Rodriguez-Velasco M.L., Otero A., Vieites J.M., Cabado A.G. (2012). A comparative study for PSP toxins quantification by using MBA and HPLC official methods in shellfish. Toxicon.

[104] Turner A.D., Lewis A.M., Rourke W.A., Higman W.A. (2014). Interlaboratory comparison of two AOAC liquid chromatographic fluorescence detection methods for paralytic shellfish toxin analysis through characterization of an oyster reference material. J. AOAC Int..

[105] Turner A.D., Broadwater M., Van Dolah F. (2018). Use of the receptor binding assay for the determination of paralytic shellfish poisoning toxins in bivalve molluscs from Great Britain and the assessment of method performance in oysters. Toxicon.

[106] Urbina M.A., Luna-Jorquera G., Thiel M., Acuna-Ruz T., Amenabar M.A., Andrade C., Ahrendt C., Castillo C., Chevallier A., Cornejo-D’Ottone M. (2020). A country’s response to tackling plastic pollution in aquatic ecosystems: The Chilean way. Aquat. Conserv..

[107] Aune T., Ramstad H., Heinenreich B., Landsverk T., Waaler T., Eggas E., Julshamn K. (1998). Zinc accumulation in oysters giving mouse deaths in paralytic shellfish poisoning bioassay. J. Shellfish Res..

[108] Vale P., de Sampayo M.A. (2001). Determination of paralytic shellfish toxins in Portuguese shellfish by automated pre-column oxidation. Toxicon.

[109] Velez P.P., Sierralta J., Alcayaga C., Fonseca M., Loyola H., Johns D.C., Tomaselli G.F., Marban E., Suarez-Isla B.A. (2001). A functional assay for paralytic shellfish toxins that uses recombinant sodium channels. Toxicon.

[110] Ruberu S.R., Langlois G.W., Masuda M., Kittredge C., Kasum Perera S., Kudela R.M. (2017). Receptor binding assay for the detection of paralytic shellfish poisoning toxins: Comparison to the mouse bioassay and applicability under regulatory use. Food Addit. Contam. Part A.

[111] Fast M.D., Cembella A.D., Ross N.W. (2006). In vitro transformation of paralytic shellfish toxins in the clams *Mya arenaria* and *Protothaca staminea*. Harmful Algae.

[112] Artigas M.L., Vale P.J.V., Gomes S.S., Bothelo M.J., Rodrigues S.M., Amorim A. (2007). Profiles of paralytic shellfish poisoning toxins in shellfish from Portugal explained by carbamoylase activity. J. Chromatogr. A.

[113] Turner A.D., Lewis A.M., O’Neil A., Hatfield R.G. (2013). Transformation of paralytic shellfish poisoning toxins in UK surf clams (*Spisula solida*) for targeted production of reference materials. Toxicon.

[114] Hignutt E. (2014). Suitability of postcolumn oxidation liquid chromatography method AOAC 2011.02 for monitoring paralytic shellfish toxins in Alaskan shellfish—Initial pilot study versus mouse bioassay and in-house validation. J. AOAC Int..

[115] Rourke W.A., Murphy C.J., Pitcher G., Van de Riet J.M., Burns B.G., Thomas K.M., Quilliam M.A. (2008). Rapid postcolumn methodology for determination of paralytic shellfish toxins in shellfish tissue. J. AOAC Int..

[116] Franca S., Alvito P., Sousa I., Gago A., Rodrıguez-Vasquez J.A., Leao J.M., Comesana M., Thibault P., Burdaspal P., Bustos J., Yasumoto T., Oshima Y., Fukuyo Y. (1996). The toxin profile of some PSP toxin producing dinoflagellates occurring in Portuguese coastal waters as determined by alternative analytical methods. Harmful and Toxic Algal Blooms.

[117] Costa P.R., Botelho M.J., Lefebvre K.A. (2010). Characterization of paralytic shellfish toxins in seawater and sardines (*Sardina pilchardus*) during blooms of *Gymnodinium catenatum*. Hydrobiologia.

[118] Costa P.R., Pereira P., Guilherme S., Barata M., Nicolau L., Santos M.A., Pacheco M., Pousao-Ferreira P. (2012). Biotransformation modulation and genotoxicity in white seabream upon exposure to paralytic shellfish toxins produced by *Gymnodinium catenatum*. Aquat. Toxicol..

[119] Costa P.R., Moita T., Rodrigues S.M. (2014). Estimating the contribution of N-sulfocarbamoyl paralytic shellfish toxin analogs GTX6 and C3+4 to the toxicity of mussels (*Mytilus galloprovincialis*) over a bloom of *Gymnodinium catenatum*. Harmful Algae.

[120] Oshiro M., Pham L., Csuti D., Dodd M., Inami G.B., Brenden R.A. (2006). Paralytic shellfish poisoning surveillance in Californica using the Jellett Rapid PSP test. Harmful Algae.

[121] DeGrasse S., Conrad S., DiStefano P., Vanegas C., Wallace D., Jensen P., Hickey J.M., Cenci F., Pitt J., Deardorff D. (2014). Onboard screening dockside testing as a new means of managing paralytic shellfish poisoning risks in federally closed waters. Deep Sea Res. Part II Top. Stud. Oceanogr..

[122] Wong C.-K., Hung P., Ng E.A.L., Lee K.L.H., Wong G.T.C., Kam K.-M. (2010). Operational application of a rapid antibody-based detection assay for first line screening of paralytic shellfish toxins in shellfish. Harmful Algae.

[123] Dennison N., Anderson D.B. The 3 “R”S approach to marine biotoxin testing in the UK. AATEX 14, special issue, 757–761. Proceedings of the 6th World Congress on Alternatives and Animal Use in the Life Sciences.

[124] Guy A.L., Griffin G. (2009). Adopting alternatives for the regulatory monitoring of shellfish for paralytic shellfish poisoning in Canada: Interface between federal regulators, science and ethics. Regul. Toxicol. Pharmacol..

[125] Hatfield R.G., Punn R., Algoet M., Turner A.D. (2016). A rapid method for the analysis of paralytic shellfish toxins utilising standards pressure HPLC: Refinement of AOAC 2005.06. J. AOAC Int..

[126] ISSC (2020). Interstate Shellfish Sanitation Conference: Single Laboratory Validation Protocol for Method Approval (Iii). http://www.issc.org/laboratory-method-references.

[127] EFSA (2009). Marine biotoxins in shellfish—Saxitoxin group. Scientific opinion of the panel on contaminants in the food chain. European Food Safety Authority. EFSA J..

[128] Laycock M.V., Kralovec J., Richards R.C. (1995). Some in vitro chemical interconversions of paralytic shellfish poisoning (PSP) toxins useful in the preparation of analytical standards. J. Mar. Biotechnol..

[129] Turner A.D., Stubbs B., Coates L., Dhanji-Rapkova M., Hatfield R.G., Lewis A.M., Rowland-Pilgrim S., O’Neil A., Stubbs P., Ross S. (2014). Variability of paralytic shellfish toxin occurrence and profiles in bivalve molluscs from Great Britain from official control monitoring as determined by pre-column oxidation liquid chromatography and implications for applying immunochemical tests. Harmful Algae.

