# Supplementary material for: Application of Six Detection Methods for Analysis of Paralytic Shellfish Toxins in Shellfish from Four Regions within Latin America
Source: Mar Drugs. 2020 Dec 3;18(12):616. doi: 10.3390/md18120616 (PMC7761785; doi:10.3390/md18120616)

Table S1. Summary of PST toxicities determined by MBA, PreCOX, PCOX, LC-MS/MS and RBA in Latin American shellfish samples, with Scotia LFA results summarised according to both visual and automated assessment including LFA scan number. (v) = viscera only; (s) = siphon only; (w) = whole tissues; nd = not detected; Pos = positive LFA results; Neg = negative LFA result; Scan value = numerical output from scanner (no units); Sample id prefix denote country of origin: A = Argentina, C = Chile, M = Mexico, U = Uruguay. Blank cells infer no test conducted. Yellow shaded cells highlight extreme outliers (more than order of magnitude difference compared with mean of other results)

			Total toxicities in µg STX eq/kg					Scotia LFA		
Sample	Shellfish	Species	MBA	PreCOX	PCOX	LC-MS/MS	RBA	Visual	Auto	Scan value
A152	Clam	*A. antiqua*	820	558	465	932	1233	Pos	Pos	0.03
A153	Clam	*A. antiqua*	1450	2120	1120	2090	5144	Pos	Pos	0.01
A166	Clam	*A. antiqua*		723	731	785		Pos	Pos	0.02
A107	Clam	*D. hanleyanus*	nd	nd		nd		Neg	Neg	1.03
A108	Clam	*D. hanleyanus*	nd	nd		39		Neg	Neg	0.96
A112	Clam	*D. hanleyanus*	nd	104		1		Neg	Neg	0.81
A113	Clam	*D. hanleyanus*		1639		nd				
A117	Clam	*D. hanleyanus*	nd	9				Neg	Neg	0.91
A119	Clam	*D. hanleyanus*	nd	5		470				
A130	Clam	*D. hanleyanus*	nd	11		nd		Neg	Neg	0.98
A154	Clam	*D. hanleyanus*	810	831	1747	1471	712	Pos		
A157	Clam	*D. hanleyanus*		117	100	171	110	Pos	Pos	0.23
A172	Clam	*D. hanleyanus*	nd	nd		5	nd	Pos		
A177	Clam	*D. hanleyanus*	nd	nd		nd		Neg	Neg	0.98
A189	Clam	*D. hanleyanus*	nd	nd		nd				
A214	Clam	*D. hanleyanus*	nd	nd		nd		Neg	Neg	0.8
A46	Clam	*M. mactroides*	1297	1359	2724	3483	1688	Pos		
A47	Clam	*M. mactroides*	1045	639	428	815	599	Pos		
A48	Clam	*M. mactroides*	1987	3484	1456	5286	2852	Neg		
A58	Clam	*M. mactroides*	1066	1290		1305		Pos		
A118	Clam	*M. mactroides*	nd	5		192				
A155	Clam	*M. mactroides*	640	553	204	902	285			
A156	Clam	*M. mactroides*	640	497	239	1729	626			
A159	Clam	*M. mactroides*		581	201	1000	482			
A160	Clam	*M. mactroides*	490	525	251	1301	759	Pos		
A174	Clam	*M. mactroides*	nd	nd		4	nd	Neg	Neg	0.9
A176	Clam	*M. mactroides*	nd	nd		1		Neg	Neg	0.92
A180	Clam	*M. mactroides*	nd	nd		4		Neg	Neg	0.9
A181	Clam	*M. mactroides*	nd	nd		23				
A182	Clam	*M. mactroides*	nd	nd		nd				
A183	Clam	*M. mactroides*	nd	nd				Pos	Neg	0.68
A184	Clam	*M. mactroides*	nd	nd		56				
A185	Clam	*M. mactroides*	nd	nd		8		Neg	Neg	0.81
A186	Clam	*M. mactroides*	nd	nd		1				
A187	Clam	*M. mactroides*	nd	nd		nd		Neg	Neg	1
A190	Clam	*M. mactroides*	nd	10		17				
A191	Clam	*M. mactroides*		nd		200	nd			
A198	Clam	*M. mactroides*	nd	nd		nd		Neg	Neg	0.97
A204	Clam	*M. mactroides*	920	238		852		Pos	Pos	0.26
A205	Clam	*M. mactroides*	380	174		417				
A206	Clam	*M. mactroides*		nd		1		Neg	Neg	0.95
A213	Clam	*M. mactroides*		442		2				
A219	Clam	*M. mactroides*	370	490		206	168	Pos	Pos	0.2
A77	Limpet	*Unknown*	372	238		1488	1775			
A67	Squid	*I. argentinus*	517	549		326	464	Pos		
A105	Squid (v)	*I. argentinus*		588		661	414	Pos	Pos	0.01
A122	Mussels	*A. ater*	nd	282		191	625	Pos	Pos	0.02
A123	Mussels	*A. ater*	nd	255		130				
A120	Mussels	*B. rodriguezii*	nd	11		197		Pos	Pos	0.49
A158	Mussels	*B. rodriguezii*	1007	226		382	316	Pos	Pos	0.06
A175	Mussels	*B. rodriguezii*	nd	nd		18	nd			
A178	Mussels	*B. rodriguezii*	nd	nd		2				

A179	Mussels	*B. rodriguezii*	nd	nd		19				
A197	Mussels	*B. rodriguezii*	710	447	661	535	216	Pos	Pos	0.1
A203	Mussels	*B. rodriguezii*		567	119	386		Pos	Pos	0.28
A215	Mussels	*B. rodriguezii*		25		47				
A218	Mussels	*B. rodriguezii*	880	656		1377	489			
A1	Mussels	*M. edulis*		2645	4065	2102		Pos		
A2	Mussels	*M. edulis*	18215	9057	8191	7310		Pos		
A3	Mussels	*M. edulis*	14446	21709	16642	24139		Pos		
A4	Mussels	*M. edulis*	41361	51670		51468	33591	Pos		
A5	Mussels	*M. edulis*	760	918	421	480	859			
A6	Mussels	*M. edulis*	306033	218375		206335				
A7	Mussels	*M. edulis*	187264	165288	21484	211897				
A8	Mussels	*M. edulis*	295957	281238	10248	402945	239633	Pos		
A9	Mussels	*M. edulis*	129747	166024	43767	193760	105057	Invalid		
A10	Mussels	*M. edulis*	173858	126593	67098	172308		Pos		
A11	Mussels	*M. edulis*	144818	171718	16958	201695		Pos		
A12	Mussels	*M. edulis*	162665	117781	2409	147802	74058	Pos		
A13	Mussels	*M. edulis*	170274	180558	5249					
A15	Mussels	*M. edulis*	18975	7361	1354	6374				
A16	Mussels	*M. edulis*	12056	9207	3190	8678		Pos		
A17	Mussels	*M. edulis*	18232	13352	7962	10423		Pos		
A18	Mussels	*M. edulis*	25981	15195	5298	13958				
A19	Mussels	*M. edulis*	33379	11371	11178	11321		Pos		
A23	Mussels	*M. edulis*	13030	8363	6777	6012		Pos		
A24	Mussels	*M. edulis*	12760	5342	5134	7913		Pos		
A33	Mussels	*M. edulis*	15700	4566	4701	4978		Pos		
A34	Mussels	*M. edulis*	14256	8562	2029	7932				
A37	Mussels	*M. edulis*	2709	2388	1315	3881	3227	Pos		
A39	Mussels	*M. edulis*	2922	906	1237	1700	1446	Pos		
A44	Mussels	*M. edulis*	2282	1437	565	1890		Pos		
A50	Mussels	*M. edulis*	25848	18954	9540	19352		Pos		
A51	Mussels	*M. edulis*	305900	296068	13014	198063	249075	Pos		
A57	Mussels	*M. edulis*	39809	15118	8155	16745		Pos		
A59	Mussels	*M. edulis*	7713	9898	5354	13634		Pos		
A60	Mussels	*M. edulis*	65643	30474	7982	35232		Pos		
A61	Mussels	*M. edulis*	100563	53196	32138	54655		Pos		
A62	Mussels	*M. edulis*	94884	64688		61461	39286			
A63	Mussels	*M. edulis*	57004	23428	26375	21977		Pos		
A64	Mussels	*M. edulis*	134858	645		941	1526			
A65	Mussels	*M. edulis*		78845	54625	74467		Pos		
A66	Mussels	*M. edulis*	284764	267324	45051	271239	271108	Pos		
A69	Mussels	*M. edulis*		4109	2896	4264		Pos		
A75	Mussels	*M. edulis*		6858	3094	6136		Pos		
A76	Mussels	*M. edulis*	31922	768		2489		Pos		
A83	Mussels	*M. edulis*	707	1453	1038	1626	1381	Pos		
A84	Mussels	*M. edulis*	1803	3180	2029	4590		Pos		
A85	Mussels	*M. edulis*		1590						
A86	Mussels	*M. edulis*		361						
A88	Mussels	*M. edulis*		30075	18439	89911				
A93	Mussels	*M. edulis*	9100	5255	2573	2837				
A94	Mussels	*M. edulis*	nd	1159	438	943				
A96	Mussels	*M. edulis*	980	1666	793	1502				
A97	Mussels	*M. edulis*	nd	36		30				
A98	Mussels	*M. edulis*	nd	36		511				
A101	Mussels	*M. edulis*		7526	2684	7931				
A110	Mussels	*M. edulis*		1639	652	1292		Pos	Pos	0.05
A111	Mussels	*M. edulis*		621		1182	1858	Pos	Pos	0.03
A121	Mussels	*M. edulis*	nd	39	205	21		Neg	Neg	0.96
A124	Mussels	*M. edulis*	nd	1021	47	1071		Pos	Pos	0.01
A136	Mussels	*M. edulis*	890	110		577	495			
A137	Mussels	*M. edulis*	820	214		493	397	Pos	Pos	0.15
A151	Mussels	*M. edulis*	14210	522	1286					
A163	Mussels	*M. edulis*		865	427					

A164	Mussels	*M. edulis*		1713	526	3765		Pos	Pos	0.35
A165	Mussels	*M. edulis*	600	530		425	627			
A168	Mussels	*M. edulis*	186	2329	730	3653	3157	Pos	Pos	0.03
A169	Mussels	*M. edulis*	67	162		498				
A170	Mussels	*M. edulis*	36	123		328				
A171	Mussels	*M. edulis*		nd		82				
A188	Mussels	*M. edulis*		10		74	102			
A192	Oysters	*C. gigas*		nd		22		Neg	Neg	0.92
A193	Oysters	*C. gigas*		nd		nd				
A194	Oysters	*C. gigas*		nd		nd				
A195	Oysters	*C. gigas*		nd		nd		Neg	Neg	0.81
A196	Oysters	*C. gigas*		nd						
A199	Oysters	*C. gigas*		nd		nd				
A200	Oysters	*C. gigas*		nd		48				
A201	Oysters	*C. gigas*		nd		14				
A222	Oysters	*C. gigas*		nd		nd		Neg	Neg	0.89
A224	Oysters	*C. gigas*		nd		nd		Neg	Neg	0.87
A225	Oysters	*C. gigas*		nd		40		Neg	Neg	0.92
A14	Scallops	*A.tehuelche*	7606	4138		6338	4127			
A20	Scallops	*A.tehuelche*	616	530	523	858	614			
A21	Scallops	*A.tehuelche*	39047	3750	8835	14505	9549			
A22	Scallops	*A.tehuelche*	23765	16326	11178	16782				
A25	Scallops	*A.tehuelche*	11366	4865	5785	8734				
A26	Scallops	*A.tehuelche*	1064	708	534	1309	1064			
A32	Scallops	*A.tehuelche*	3996	2987		2473	1607			
A90	Scallops	*A.tehuelche*		1078	616	2114				
A91	Scallops	*A.tehuelche*	nd	3597	5093	9016				
A92	Scallops	*A.tehuelche*	nd	29		101				
A139	Scallops	*A.tehuelche*	360	15		1		Neg	Neg	0.92
A140	Scallops	*A.tehuelche*	730	4910						
A144	Scallops	*A.tehuelche*	22640	6537	1784	14252		Pos	Pos	0.04
A145	Scallops	*A.tehuelche*	21260	1465						
A146	Scallops	*A.tehuelche*	19660	6497	2667	9001		Pos	Pos	0.02
A147	Scallops	*A.tehuelche*	17320	2972		10861				
A161	Scallops	*A.tehuelche*	nd	169		202				
A162	Scallops	*A.tehuelche*	nd	162		359				
A95	Scallops	*Z.patagonica*	980	932	3216	3586	5360			
A102	Scallops	*Z.patagonica*		1017	1591	1659	5589	Pos	Pos	0.02
A104	Scallops	*Z.patagonica*		506	564	1547	1224	Pos	Pos	0.22
A125	Scallops	*Z.patagonica*	nd	943	1872					
A126	Scallops	*Z.patagonica*	nd	1795						
A127	Scallops	*Z.patagonica*	nd	1287	3039					
A129	Scallops	*Z.patagonica*		179		390	459	Pos	Pos	0.04
A131	Scallops	*Z.patagonica*	3140	1033	1519					
A132	Scallops	*Z.patagonica*	2170	2838	1920					
A133	Scallops	*Z.patagonica*	2390	1105	1897					
A134	Scallops	*Z.patagonica*	2160	1450	724					
A135	Scallops	*Z.patagonica*	2470	1390	2169					
A138	Scallops	*Z.patagonica*	9570	2304	2794					
A141	Scallops	*Z.patagonica*	450	3392		4		Pos	Pos	0.43
A142	Scallops	*Z.patagonica*	480	1537		13	89	Pos	Pos	0.24
A143	Scallops	*Z.patagonica*	370	1751		425		Pos	Pos	0.33
A149	Scallops	*Z.patagonica*	2430	503	683	1321		Pos	Pos	0.09
A150	Scallops	*Z.patagonica*	750	387		1124	802			
A173	Scallops	*Z.patagonica*	1890	166						
A208	Scallops	*Z.patagonica*	1210	nd		3				
A209	Scallops	*Z.patagonica*		185		168				
A216	Scallops	*Z.patagonica*	nd	nd		nd				
A217	Scallops	*Z.patagonica*	nd	nd		3		Neg	Neg	0.73
A220	Scallops	*Z.patagonica*	nd	nd		2		Pos	Neg	0.64
A221	Scallops	*Z.patagonica*	nd	nd		2				
A109	Shrimp	*Unknown*	nd	110						
A31	Snail	*A. beckii*	1159	2031	2092	1085	2324	Pos		

A78	Snail	*A. beckii*	1934	3129	3662			Pos		
A81	Snail	*A. beckii*		3107	2794	1283		Pos		
A100	Snail	*A. beckii*		1325	1617	799	2310			
A103	Snail	*A. beckii*		1666	1956					
A148	Snail	*A. beckii*	990	701	825	359	1358	Pos	Pos	0.04
A27	Snail	*Zidona dufresnei*	22468	9250	2696	4599		Pos		
A28	Snail	*Zidona dufresnei*	20727	28532	19954	8539	25679			
A29	Snail	*Zidona dufresnei*	31314	18275	23453	13153		Pos		
A30	Snail	*Zidona dufresnei*	7317	5226	6675	2188	5851	Pos		
A35	Snail	*Zidona dufresnei*	1986	1791	1786	1230	4778	Pos		
A36	Snail	*Zidona dufresnei*	2206	2098	1258	1070	2115	Pos		
A38	Snail	*Zidona dufresnei*	3164	3793	3726	2599		Pos		
A40	Snail	*Zidona dufresnei*	1759	1840	1188	1126	1969	Pos		
A41	Snail	*Zidona dufresnei*	1911	3196	2452	2026		Pos		
A42	Snail	*Zidona dufresnei*	2010	273		147	148	Pos		
A43	Snail	*Zidona dufresnei*	1820	3076	3253	2606		Pos		
A45	Snail	*Zidona dufresnei*	1615	2322	2722	1856		Pos		
A49	Snail	*Zidona dufresnei*	2662	3608	4855	3544		Pos		
A52	Snail	*Zidona dufresnei*	2367	1713	1966	1092		Pos		
A53	Snail	*Zidona dufresnei*	2242	1221	960	2857				
A54	Snail	*Zidona dufresnei*	2660	3914	4599	2561	6425	Neg		
A55	Snail	*Zidona dufresnei*	3365	2348	2690	2824		Pos		
A56	Snail	*Zidona dufresnei*	2557	4835		3488	10034	Pos		
A70	Snail	*Zidona dufresnei*	3830	3418	4360	2139	5199	Pos		
A71	Snail	*Zidona dufresnei*		945	969	686	1856	Pos		
A72	Snail	*Zidona dufresnei*		10744	14399	9707		Pos		
A73	Snail	*Zidona dufresnei*	17102	21622	768	18400	39695	Invalid		
A74	Snail	*Zidona dufresnei*	1520	2168	3278	2006		Pos		
A79	Snail	*Zidona dufresnei*	1554	3107	3438	2403		Pos		
A80	Snail	*Zidona dufresnei*		1768	2295	1153				
A82	Snail	*Zidona dufresnei*	2101	3354	4647	2206	6725			
A99	Snail	*Zidona dufresnei*	nd	161		99				
A106	Snail	*Zidona dufresnei*		60	17	39	121			
A114	Snail	*Zidona dufresnei*	nd	74		38		Neg	Neg	0.67
A115	Snail	*Zidona dufresnei*	nd	115	39	92	227	Pos	Neg	0.63
A116	Snail	*Zidona dufresnei*	nd	62		25		Pos	Neg	0.79
A207	Snail	*Zidona dufresnei*		442	346	257	402			
A210	Snail	*Zidona dufresnei*		490	448	163	609			
A211	Snail	*Zidona dufresnei*		475		204		Pos	Pos	0.04
A212	Snail	*Zidona dufresnei*		206		191				
C1	Clam	*Gari solida*	1140	446	149	20	327	Pos		
C2	Clam	*V. antiqua*	4080	156		91	81	Pos		
C4	Clam	*V. antiqua*	120040	5287	5356	4552	4841	Pos		
C20	Clam	*V. antiqua*	400	239	213	322		Pos		
C21	Clam	*V. antiqua*	970	322	293	452	320	Pos		
C22	Clam	*V. antiqua*	910	422	11	1012		Pos		
C23	Clam	*V. antiqua*	300	98		96		Pos		
C5	Mussels	*M. chilensis*	1079	1148	1335	318	1359	Pos		
C6	Mussels	*M. chilensis*	850	1430		468		Pos		
C8	Mussels	*M. chilensis*	350	2247	1068	682	2628	Pos		
C9	Mussels	*M. chilensis*	4220	1471		330		Pos		
C11	Mussels	*M. chilensis*	15890	5992	5795	5166		Pos		
C12	Mussels	*M. chilensis*	2330	996	359	833		Pos		
C13	Mussels	*M. chilensis*	1530	329	951	333		Pos		
C14	Mussels	*M. chilensis*	2210	498	39	493	535	Pos		
C15	Mussels	*M. chilensis*	710	413		303		Pos		
C16	Mussels	*M. chilensis*	14750	5043	4283	5073	7047	Pos		
C17	Mussels	*M. chilensis*	39750	19129	17436	17772		Pos		
C18	Mussels	*M. chilensis*	6500	3344		3741		Pos		
C19	Mussels	*M. chilensis*	8840	2141	56	2599	2961	Pos		
C24	Mussels	*M. chilensis*	280	114		161		Pos		
C25	Mussels	*M. chilensis*	1330	1742	694	1786	2404	Pos		
C27	Mussels	*M. chilensis*	410	294	454	328		Pos		

C28	Mussels	*M. chilensis*	1660	461	420	452	550	Pos
C29	Mussels	*M. chilensis*	1150	808	547	1075		Pos
C30	Mussels	*M. chilensis*	320	116		156		Pos
C31	Mussels	*M. chilensis*	601	280	316	399		Pos
C32	Mussels	*M. chilensis*	1000	770	421	973	1172	Pos
C33	Mussels	*M. chilensis*	313	129		111		Pos
C34	Mussels	*M. chilensis*	329	389	527	309		Pos
C35	Mussels	*M. chilensis*	416	323	321	285		Pos
C36	Mussels	*M. chilensis*	693	367	380	221		Pos
C37	Mussels	*M. chilensis*	323	271	430	249		Pos
C26	Oysters	*C. gigas*	1070	870	523	859	1016	Pos
C3	Scallops	*A. purpuratus*	356	316	291	84	472	Pos
C7	Scallops	*A. purpuratus*	330	545	638	138		Pos
C10	Scallops	*A. purpuratus*	321	382	387	205	530	Pos
M12-S12	Geoduck (s)	*P. generosa*	nd	37				Pos
M15-S15	Geoduck (s)	*P. generosa*	nd	15				Pos
M17-S18	Geoduck (s)	*P. generosa*	nd	nd		107	62/nd	Neg
M19-S21	Geoduck (s)	*P. generosa*	nd	11				Pos
M21-S24	Geoduck (s)	*P. generosa*	nd	116		61	nd	Neg
M22-S27	Geoduck (s)	*P. generosa*	nd	19				Pos
M25-S30	Geoduck (s)	*P. generosa*	nd	19				Pos
M28-S36	Geoduck (s)	*P. generosa*	nd	21				Pos
M30-S39	Geoduck (s)	*P. generosa*		52				Neg
M32-S42	Geoduck (s)	*P. generosa*		54				Neg
M35-S48	Geoduck (s)	*P. generosa*		5				Pos
M37-S51	Geoduck (s)	*P. generosa*		30				Neg
M39-S57	Geoduck (s)	*P. generosa*		7				Neg
M3-S3	Geoduck (s)	*P. generosa*	nd	43		113		Pos
M41-S69	Geoduck (s)	*P. generosa*		55				Pos
M43-S72	Geoduck (s)	*P. generosa*		20				Neg
M45-S96	Geoduck (s)	*P. generosa*		6				Neg
M47-S105	Geoduck (s)	*P. generosa*	nd	87		97	nd	Pos
M49-S111	Geoduck (s)	*P. generosa*		26				Neg
M51-S114	Geoduck (s)	*P. generosa*		43				Neg
M52-S117	Geoduck (s)	*P. generosa*		36				Neg
M54-S120	Geoduck (s)	*P. generosa*		80				Neg
M56-S130	Geoduck (s)	*P. generosa*		nd				Neg
M58-S140	Geoduck (s)	*P. generosa*		10		125		Neg
M60-S143	Geoduck (s)	*P. generosa*		34				
M62-S149	Geoduck (s)	*P. generosa*		9				Neg
M64-S152	Geoduck (s)	*P. generosa*		16				Neg
M66-S158	Geoduck (s)	*P. generosa*		5				Pos
M68-S161	Geoduck (s)	*P. generosa*		6				Neg
M6-S6	Geoduck (s)	*P. generosa*	nd	30				Neg
M70-S33	Geoduck (s)	*P. generosa*	nd	nd				Neg
M71-S45	Geoduck (s)	*P. generosa*		18		30	nd	Neg
M72-S93	Geoduck (s)	*P. generosa*	nd	27				Pos
M9-S9	Geoduck (s)	*P. generosa*	nd	123		199	69/nd	Pos
M10-C10	Geoduck (w)	*P. generosa*	nd	110				Pos
M13-C13	Geoduck (w)	*P. generosa*		104		196		Pos
M1-C1	Geoduck (w)	*P. generosa*	448	492		394	102	Pos
M23-C28	Geoduck (w)	*P. generosa*		78		133		Pos
M4-C4	Geoduck (w)	*P. generosa*	590	362		280	183	Pos
M7-C7	Geoduck (w)	*P. generosa*	nd	254		543		Pos
M11-V11	Geoduck (w)	*P. generosa*	596	287		846	149	Pos
M14-V14	Geoduck (w)	*P. generosa*	772	295		935	238	Pos
M16-V17	Geoduck (w)	*P. generosa*	nd	182		446		Pos
M18-V20	Geoduck (w)	*P. generosa*	nd	150		947		Pos
M20-V23	Geoduck (w)	*P. generosa*	nd	292		483	105	Pos
M24-V29	Geoduck (w)	*P. generosa*	504	800		1024	138	Pos
M26-V32	Geoduck (w)	*P. generosa*	nd	83				Pos
M27-V35	Geoduck (w)	*P. generosa*	512	366		852	303	Pos
M29-V38	Geoduck (w)	*P. generosa*	nd	107				Pos

M2-V2	Geoduck (w)	*P. generosa*	2260	363		1333	574	Pos
M31-V41	Geoduck (w)	*P. generosa*	nd	165		408	116	Pos
M33-V44	Geoduck (w)	*P. generosa*	nd	89		112		Neg
M34-V47	Geoduck (w)	*P. generosa*	nd	136				Pos
M36-V50	Geoduck (w)	*P. generosa*	nd	99				Pos
M38-V56	Geoduck (w)	*P. generosa*	nd	37				Pos
M40-V68	Geoduck (w)	*P. generosa*	nd	129				Pos
M42-V71	Geoduck (w)	*P. generosa*	nd	85				Pos
M44-V92	Geoduck (w)	*P. generosa*	863	437	140		153	Pos
M46-V104	Geoduck (w)	*P. generosa*	540	518	425		220	Pos
M48-V110	Geoduck (w)	*P. generosa*		152		108		Pos
M50-V113	Geoduck (w)	*P. generosa*		117				Pos
M53-V119	Geoduck (w)	*P. generosa*		129				Pos
M55-V129	Geoduck (w)	*P. generosa*		nd		12		Neg
M57-V139	Geoduck (w)	*P. generosa*		130				Pos
M59-V142	Geoduck (w)	*P. generosa*		168		177		Pos
M5-V5	Geoduck (w)	*P. generosa*	1293	350		723	295	Pos
M61-V148	Geoduck (w)	*P. generosa*		118				Pos
M63-V151	Geoduck (w)	*P. generosa*		159				Pos
M65-V157	Geoduck (w)	*P. generosa*		7				Pos
M67-V160	Geoduck (w)	*P. generosa*		20				Pos
M69-V26	Geoduck (w)	*P. generosa*	440	119		393	153	Pos
M73-V95	Geoduck (w)	*P. generosa*	384	250		361	98	Pos
M74-V116	Geoduck (w)	*P. generosa*		120				Pos
M8-V8	Geoduck (w)	*P. generosa*	4120	1582	1277	3680	792	Pos
U9	Clam	*D. hanleyanus*	4000	193	268	553	240	Pos
U11	Clam	*D. hanleyanus*	799	209	81	465		Pos
U13	Clam	*D. hanleyanus*	2470	982	368		27389	Pos
U18	Clam	*D. hanleyanus*	630	520	249	1018	519	Pos
U19	Clam	*D. hanleyanus*	nd	10		50	nd	Neg
U1	Mussels	*M. edulis*	1670	546	337		394	Pos
U2	Mussels	*M. edulis*	799	370	513			Pos
U3	Mussels	*M. edulis*	1160	2085	3047	2040		Pos
U4	Mussels	*M. edulis*	830	1680	559	2180	1335	Pos
U5	Mussels	*M. edulis*		4439	3908	6632		Pos
U6	Mussels	*M. edulis*	799	326		406		Pos
U7	Mussels	*M. edulis*	1960	453	27	697	1242	Pos
U8	Mussels	*M. edulis*		1022	1774	2053		Pos
U10	Mussels	*M. edulis*	1970	270		415	176	Pos
U12	Mussels	*M. edulis*	2900	1426	1087			
U14	Mussels	*M. edulis*	3090	7280	3439	14029	5345	Pos
U15	Mussels	*M. edulis*	17830	25580	38425	38804		Pos
U16	Mussels	*M. edulis*	10300	2004	3229	2570	1939	Pos
U17	Mussels	*M. edulis*	11500	560	2264	4241	3344	Pos
U20	Mussels	*M. edulis*	nd	10		4		Neg

Table S2. Summary of total PST (µg STX eq/kg) determined by LC-MS/MS, associated toxin profiles (in terms of proportion saxitoxin equivalents), cluster types and total number of toxin analogue detections for all Latin American shellfish samples exhibiting total PST > 16 µg STX eq/kg

Sample	Total PST	GTX 1&4	dcNEO	NEO	dcGTX 2&3	C 1&2	dcSTX	GTX 2&3	GTX 5	STX	GTX6	doSTX	C3&4	dcGTX1&4	M toxins	Cluster
A1	2102	0.00	0.00	0.00	0.00	0.00	0.12	0.00	0.00	0.88	0.00	0.00	0.00	0.00	0.00	1
A2	7310	0.82	0.00	0.01	0.00	0.01	0.00	0.15	0.00	0.01	0.00	0.00	0.00	0.00	0.00	2
A3	24139	0.96	0.00	0.00	0.00	0.00	0.00	0.04	0.00	0.00	0.00	0.00	0.00	0.00	0.00	2
A4	51468	0.93	0.00	0.01	0.00	0.00	0.00	0.05	0.00	0.01	0.00	0.00	0.00	0.00	0.00	2
A5	480	0.42	0.00	0.09	0.00	0.00	0.00	0.11	0.00	0.38	0.00	0.00	0.00	0.00	0.01	3
A6	206335	0.92	0.00	0.00	0.00	0.02	0.00	0.04	0.00	0.00	0.00	0.00	0.00	0.00	0.01	2
A7	211897	0.92	0.00	0.00	0.01	0.02	0.00	0.05	0.00	0.00	0.00	0.00	0.00	0.00	0.01	2
A8	402945	0.91	0.00	0.00	0.00	0.02	0.00	0.06	0.00	0.00	0.00	0.00	0.00	0.00	0.01	2
A9	193760	0.91	0.00	0.00	0.00	0.01	0.00	0.06	0.00	0.00	0.00	0.00	0.00	0.00	0.01	2
A10	172308	0.87	0.00	0.00	0.01	0.02	0.00	0.10	0.00	0.00	0.00	0.00	0.00	0.00	0.00	2
A11	201695	0.90	0.00	0.00	0.00	0.02	0.00	0.07	0.00	0.00	0.00	0.00	0.00	0.00	0.01	2
A12	147802	0.89	0.00	0.00	0.00	0.02	0.00	0.07	0.00	0.00	0.00	0.00	0.00	0.00	0.01	2
A14	6338	0.88	0.00	0.02	0.00	0.00	0.00	0.07	0.00	0.01	0.00	0.00	0.00	0.00	0.02	2
A15	6374	0.95	0.00	0.00	0.00	0.01	0.00	0.03	0.00	0.00	0.00	0.00	0.00	0.00	0.01	2
A16	8678	0.96	0.00	0.00	0.00	0.01	0.00	0.02	0.00	0.00	0.00	0.00	0.00	0.00	0.00	2
A17	10423	0.96	0.00	0.00	0.00	0.01	0.00	0.02	0.00	0.00	0.00	0.00	0.00	0.00	0.01	2
A18	13958	0.96	0.00	0.00	0.00	0.01	0.00	0.02	0.00	0.00	0.00	0.00	0.00	0.00	0.00	2
A19	11321	0.92	0.00	0.01	0.00	0.01	0.00	0.06	0.00	0.00	0.00	0.00	0.00	0.00	0.00	2
A20	858	0.87	0.00	0.01	0.00	0.01	0.00	0.10	0.00	0.00	0.00	0.00	0.00	0.00	0.00	2
A21	14505	0.81	0.00	0.01	0.00	0.01	0.00	0.13	0.00	0.01	0.00	0.00	0.00	0.00	0.03	2
A22	16781	0.85	0.00	0.00	0.00	0.01	0.00	0.13	0.00	0.00	0.00	0.00	0.00	0.00	0.01	2
A23	6012	0.87	0.00	0.00	0.00	0.01	0.00	0.10	0.00	0.01	0.00	0.00	0.00	0.00	0.01	2
A24	7913	0.87	0.00	0.01	0.00	0.01	0.00	0.09	0.00	0.01	0.00	0.00	0.00	0.00	0.01	2
A25	8734	0.75	0.00	0.01	0.00	0.01	0.00	0.20	0.00	0.01	0.01	0.00	0.00	0.00	0.01	2
A26	1309	0.77	0.00	0.01	0.00	0.01	0.00	0.19	0.00	0.01	0.00	0.00	0.00	0.00	0.02	2
A27	4599	0.01	0.00	0.01	0.00	0.00	0.02	0.05	0.00	0.91	0.00	0.00	0.00	0.00	0.00	1
A28	8539	0.00	0.00	0.01	0.00	0.00	0.02	0.00	0.00	0.96	0.00	0.00	0.00	0.00	0.00	1
A29	13153	0.00	0.00	0.04	0.00	0.00	0.03	0.00	0.00	0.92	0.00	0.00	0.00	0.00	0.00	1
A30	2188	0.00	0.00	0.04	0.00	0.00	0.01	0.00	0.00	0.94	0.00	0.00	0.00	0.00	0.00	1
A31	1085	0.00	0.00	0.00	0.00	0.00	0.02	0.00	0.00	0.98	0.00	0.00	0.00	0.00	0.00	1
A32	2473	0.87	0.00	0.01	0.00	0.00	0.00	0.09	0.00	0.01	0.00	0.00	0.00	0.00	0.01	2
A33	4978	0.90	0.00	0.01	0.00	0.00	0.00	0.03	0.00	0.00	0.00	0.00	0.00	0.00	0.05	2
A34	7932	0.89	0.00	0.00	0.00	0.00	0.00	0.08	0.00	0.00	0.01	0.00	0.00	0.00	0.01	2
A35	1230	0.02	0.00	0.05	0.00	0.00	0.01	0.00	0.00	0.91	0.00	0.00	0.00	0.00	0.00	1
A36	1070	0.00	0.00	0.00	0.00	0.00	0.01	0.00	0.00	0.99	0.00	0.00	0.00	0.00	0.00	1
A37	3881	0.86	0.00	0.02	0.00	0.00	0.00	0.08	0.00	0.01	0.00	0.00	0.00	0.00	0.02	2
A38	2599	0.03	0.00	0.05	0.00	0.00	0.01	0.01	0.00	0.91	0.00	0.00	0.00	0.00	0.00	1
A39	1700	0.90	0.00	0.01	0.00	0.00	0.00	0.06	0.00	0.01	0.00	0.00	0.00	0.00	0.02	2
A40	1126	0.00	0.00	0.00	0.00	0.00	0.01	0.00	0.00	0.99	0.00	0.00	0.00	0.00	0.00	1
A41	2026	0.00	0.00	0.04	0.00	0.00	0.01	0.00	0.00	0.95	0.00	0.00	0.00	0.00	0.00	1
A42	147	0.35	0.00	0.00	0.00	0.00	0.00	0.02	0.00	0.63	0.00	0.00	0.00	0.00	0.00	1
A43	2606	0.03	0.00	0.04	0.00	0.00	0.01	0.01	0.00	0.91	0.00	0.00	0.00	0.00	0.00	1

A44	1890	0.87	0.00	0.00	0.00	0.00	0.00	0.06	0.00	0.01	0.00	0.00	0.00	0.00	0.06	2
A45	1856	0.06	0.00	0.04	0.00	0.00	0.01	0.01	0.00	0.89	0.00	0.00	0.00	0.00	0.00	1
A46	3483	0.63	0.00	0.01	0.24	0.00	0.03	0.07	0.01	0.01	0.00	0.00	0.00	0.00	0.00	2
A47	815	0.67	0.00	0.01	0.00	0.06	0.00	0.21	0.01	0.02	0.00	0.00	0.00	0.00	0.02	2
A48	5286	0.74	0.00	0.00	0.02	0.10	0.00	0.07	0.00	0.01	0.00	0.00	0.00	0.00	0.05	2
A49	3544	0.01	0.00	0.05	0.00	0.00	0.01	0.00	0.00	0.93	0.00	0.00	0.00	0.00	0.00	1
A50	19352	0.91	0.00	0.01	0.00	0.00	0.00	0.07	0.00	0.00	0.00	0.00	0.00	0.00	0.01	2
A51	198063	0.92	0.00	0.01	0.00	0.00	0.00	0.05	0.00	0.00	0.00	0.00	0.00	0.00	0.02	2
A52	1092	0.00	0.00	0.03	0.00	0.00	0.01	0.00	0.00	0.96	0.00	0.00	0.00	0.00	0.00	1
A53	2857	0.23	0.00	0.03	0.00	0.00	0.00	0.02	0.00	0.71	0.00	0.00	0.00	0.00	0.00	1
A54	2561	0.01	0.00	0.06	0.00	0.00	0.01	0.01	0.00	0.92	0.00	0.00	0.00	0.00	0.00	1
A56	3488	0.02	0.00	0.00	0.00	0.00	0.01	0.01	0.00	0.96	0.00	0.00	0.00	0.00	0.00	1
A57	16745	0.90	0.00	0.01	0.00	0.00	0.00	0.05	0.00	0.00	0.00	0.00	0.00	0.00	0.04	2
A58	1305	0.70	0.00	0.00	0.00	0.04	0.00	0.24	0.00	0.01	0.00	0.00	0.00	0.00	0.01	2
A59	13634	0.92	0.00	0.00	0.00	0.00	0.00	0.06	0.00	0.00	0.00	0.00	0.00	0.00	0.01	2
A60	35232	0.91	0.00	0.00	0.00	0.00	0.00	0.07	0.00	0.00	0.00	0.00	0.00	0.00	0.02	2
A61	54655	0.95	0.00	0.01	0.00	0.00	0.00	0.04	0.00	0.00	0.00	0.00	0.00	0.00	0.01	2
A62	61461	0.95	0.00	0.01	0.00	0.00	0.00	0.03	0.00	0.00	0.00	0.00	0.00	0.00	0.01	2
A63	21977	0.90	0.00	0.01	0.00	0.00	0.00	0.06	0.00	0.00	0.00	0.00	0.00	0.00	0.02	2
A64	941	0.42	0.00	0.30	0.00	0.00	0.00	0.13	0.01	0.12	0.00	0.00	0.00	0.00	0.02	3
A65	74467	0.62	0.00	0.16	0.00	0.01	0.00	0.13	0.04	0.04	0.00	0.00	0.00	0.00	0.00	2
A66	271239	0.92	0.00	0.01	0.00	0.01	0.00	0.04	0.00	0.00	0.01	0.00	0.00	0.00	0.02	2
A67	326	0.66	0.00	0.08	0.00	0.00	0.00	0.17	0.00	0.07	0.00	0.00	0.00	0.00	0.02	2
A69	4264	0.92	0.00	0.00	0.00	0.00	0.00	0.07	0.00	0.01	0.00	0.00	0.00	0.00	0.00	2
A70	2139	0.00	0.00	0.04	0.00	0.00	0.01	0.00	0.00	0.94	0.00	0.00	0.00	0.00	0.00	1
A71	686	0.00	0.00	0.08	0.00	0.00	0.01	0.01	0.00	0.82	0.00	0.00	0.00	0.00	0.09	1
A72	9707	0.01	0.00	0.04	0.00	0.00	0.01	0.05	0.00	0.88	0.00	0.00	0.00	0.00	0.01	1
A73	18400	0.24	0.00	0.04	0.01	0.00	0.01	0.22	0.00	0.46	0.00	0.00	0.00	0.00	0.01	3
A74	2006	0.00	0.00	0.05	0.00	0.00	0.01	0.00	0.00	0.93	0.00	0.00	0.00	0.00	0.00	1
A75	6136	0.92	0.00	0.00	0.00	0.00	0.00	0.04	0.00	0.01	0.00	0.00	0.00	0.00	0.02	2
A76	2489	0.00	0.00	0.09	0.00	0.00	0.02	0.00	0.00	0.89	0.00	0.00	0.00	0.00	0.00	1
A77	1488	0.91	0.00	0.00	0.00	0.00	0.00	0.06	0.00	0.01	0.00	0.00	0.00	0.00	0.02	2
A79	2403	0.00	0.00	0.07	0.00	0.00	0.01	0.00	0.00	0.92	0.00	0.00	0.00	0.00	0.00	1
A80	1153	0.00	0.00	0.05	0.00	0.00	0.01	0.00	0.00	0.94	0.00	0.00	0.00	0.00	0.00	1
A81	1283	0.00	0.00	0.00	0.00	0.00	0.02	0.00	0.00	0.98	0.00	0.00	0.00	0.00	0.00	1
A82	2206	0.00	0.00	0.05	0.00	0.00	0.01	0.00	0.00	0.93	0.00	0.00	0.00	0.00	0.00	1
A83	1626	0.83	0.00	0.02	0.00	0.00	0.00	0.07	0.00	0.06	0.00	0.00	0.00	0.00	0.01	2
A84	4590	0.92	0.00	0.01	0.00	0.00	0.00	0.05	0.00	0.01	0.00	0.00	0.00	0.00	0.01	2
A88	89911	0.92	0.00	0.00	0.00	0.01	0.00	0.05	0.00	0.00	0.01	0.00	0.00	0.00	0.01	2
A90	2107	0.15	0.01	0.07	0.10	0.12	0.08	0.24	0.07	0.03	0.09	0.00	0.00	0.00	0.02	3
A91	9016	0.83	0.00	0.01	0.00	0.01	0.00	0.14	0.00	0.01	0.00	0.00	0.00	0.00	0.01	2
A92	101	0.93	0.00	0.03	0.00	0.00	0.00	0.04	0.00	0.01	0.00	0.00	0.00	0.00	0.00	2
A93	2837	0.93	0.00	0.01	0.00	0.00	0.00	0.04	0.00	0.00	0.01	0.00	0.00	0.00	0.02	2
A94	943	0.89	0.00	0.00	0.00	0.01	0.00	0.08	0.00	0.01	0.00	0.00	0.00	0.00	0.01	2
A95	3586	0.14	0.00	0.00	0.01	0.01	0.00	0.77	0.01	0.05	0.00	0.00	0.00	0.00	0.01	3
A96	1502	0.72	0.00	0.00	0.00	0.01	0.00	0.25	0.00	0.01	0.00	0.00	0.00	0.00	0.01	2

A97	28	0.00	0.00	0.07	0.00	0.00	0.00	0.26	0.00	0.66	0.00	0.00	0.00	0.00	0.01	1
A98	508	0.20	0.02	0.05	0.05	0.11	0.10	0.26	0.04	0.07	0.04	0.00	0.00	0.00	0.07	3
A99	99	0.00	0.00	0.03	0.00	0.00	0.03	0.00	0.00	0.94	0.00	0.00	0.00	0.00	0.00	1
A100	799	0.00	0.00	0.00	0.00	0.00	0.01	0.00	0.00	0.99	0.00	0.00	0.00	0.00	0.00	1
A101	7931	0.92	0.00	0.00	0.00	0.00	0.00	0.07	0.00	0.00	0.00	0.00	0.00	0.00	0.00	2
A102	1659	0.49	0.00	0.37	0.00	0.00	0.00	0.08	0.01	0.05	0.00	0.00	0.00	0.00	0.00	2
A104	1547	0.44	0.00	0.01	0.01	0.01	0.00	0.50	0.01	0.02	0.00	0.00	0.00	0.00	0.01	3
A105	661	0.08	0.00	0.10	0.08	0.06	0.03	0.33	0.03	0.07	0.01	0.00	0.00	0.00	0.21	3
A106	39	0.00	0.00	0.00	0.00	0.00	0.35	0.00	0.00	0.65	0.00	0.00	0.00	0.00	0.00	1
A108	39	0.87	0.00	0.00	0.00	0.04	0.00	0.08	0.00	0.01	0.00	0.00	0.00	0.00	0.00	2
A110	1292	0.81	0.00	0.00	0.00	0.00	0.00	0.15	0.00	0.01	0.00	0.00	0.00	0.00	0.02	2
A111	1182	0.31	0.00	0.17	0.00	0.00	0.00	0.12	0.02	0.36	0.00	0.00	0.00	0.00	0.01	3
A114	38	0.00	0.00	0.03	0.00	0.00	0.07	0.00	0.00	0.90	0.00	0.00	0.00	0.00	0.00	1
A115	92	0.00	0.00	0.00	0.00	0.00	0.10	0.00	0.00	0.90	0.00	0.00	0.00	0.00	0.00	1
A116	25	0.00	0.00	0.04	0.00	0.00	0.05	0.00	0.00	0.91	0.00	0.00	0.00	0.00	0.00	1
A118	192	0.00	0.00	0.00	0.00	0.07	0.21	0.00	0.55	0.01	0.00	0.00	0.00	0.00	0.15	3
A119	470	0.00	0.00	0.00	0.02	0.00	0.97	0.00	0.00	0.01	0.00	0.00	0.00	0.00	0.00	3
A120	197	0.00	0.00	0.00	0.00	0.04	0.34	0.00	0.48	0.01	0.00	0.00	0.00	0.00	0.13	3
A121	21	0.70	0.00	0.00	0.00	0.05	0.10	0.06	0.07	0.03	0.00	0.00	0.00	0.00	0.00	2
A122	191	0.00	0.00	0.00	0.00	0.00	0.01	0.37	0.08	0.53	0.00	0.00	0.00	0.00	0.01	3
A123	130	0.00	0.00	0.00	0.00	0.00	0.01	0.22	0.04	0.73	0.00	0.00	0.00	0.00	0.00	1
A124	1062	0.16	0.00	0.07	0.00	0.00	0.00	0.22	0.03	0.52	0.00	0.00	0.00	0.00	0.00	3
A129	390	0.12	0.00	0.00	0.00	0.01	0.00	0.77	0.00	0.10	0.00	0.00	0.00	0.00	0.01	3
A136	577	0.72	0.00	0.01	0.00	0.01	0.00	0.19	0.00	0.02	0.00	0.00	0.00	0.00	0.06	2
A137	493	0.68	0.00	0.06	0.00	0.01	0.00	0.17	0.00	0.02	0.00	0.00	0.00	0.00	0.06	2
A144	14243	0.74	0.00	0.02	0.01	0.01	0.00	0.18	0.00	0.02	0.00	0.00	0.00	0.00	0.03	2
A146	8995	0.74	0.00	0.03	0.00	0.00	0.00	0.17	0.00	0.03	0.00	0.00	0.00	0.00	0.02	2
A147	10861	0.68	0.00	0.03	0.01	0.01	0.00	0.20	0.00	0.04	0.00	0.00	0.00	0.00	0.03	2
A148	359	0.00	0.00	0.00	0.00	0.00	0.03	0.00	0.00	0.97	0.00	0.00	0.00	0.00	0.00	1
A149	1321	0.47	0.00	0.01	0.01	0.01	0.00	0.42	0.00	0.04	0.01	0.00	0.00	0.00	0.02	3
A150	1124	0.49	0.00	0.01	0.01	0.01	0.00	0.44	0.01	0.03	0.00	0.00	0.00	0.00	0.00	3
A152	932	0.41	0.00	0.01	0.00	0.06	0.00	0.47	0.00	0.04	0.00	0.00	0.00	0.00	0.01	3
A153	2090	0.51	0.00	0.01	0.00	0.05	0.00	0.38	0.00	0.03	0.00	0.00	0.00	0.00	0.02	3
A154	1471	0.11	0.01	0.01	0.64	0.00	0.06	0.02	0.04	0.00	0.01	0.00	0.00	0.10	0.00	3
A155	902	0.00	0.00	0.00	0.24	0.20	0.16	0.02	0.29	0.00	0.01	0.00	0.00	0.00	0.08	3
A156	1729	0.01	0.00	0.00	0.25	0.21	0.09	0.02	0.23	0.00	0.00	0.00	0.00	0.03	0.15	3
A157	171	0.00	0.00	0.00	0.66	0.00	0.29	0.02	0.02	0.01	0.00	0.00	0.00	0.00	0.00	3
A158	382	0.00	0.05	0.01	0.26	0.11	0.36	0.02	0.07	0.00	0.02	0.00	0.00	0.00	0.10	3
A159	1000	0.01	0.01	0.00	0.23	0.24	0.16	0.01	0.20	0.01	0.00	0.00	0.00	0.02	0.11	3
A160	1301	0.01	0.00	0.00	0.30	0.19	0.09	0.04	0.19	0.00	0.01	0.00	0.00	0.03	0.13	3
A161	202	0.45	0.00	0.03	0.01	0.02	0.00	0.41	0.01	0.07	0.00	0.00	0.00	0.00	0.01	3
A162	359	0.22	0.00	0.02	0.00	0.02	0.01	0.64	0.00	0.09	0.00	0.00	0.00	0.00	0.01	3
A164	3765	0.90	0.00	0.00	0.00	0.02	0.00	0.07	0.00	0.00	0.00	0.00	0.00	0.00	0.00	2
A165	785	0.69	0.00	0.01	0.01	0.07	0.00	0.15	0.00	0.01	0.00	0.00	0.00	0.00	0.05	2
A166	1446	0.37	0.00	0.00	0.00	0.01	0.00	0.57	0.00	0.04	0.00	0.00	0.00	0.00	0.01	3
A168	3653	0.79	0.00	0.01	0.00	0.02	0.00	0.11	0.00	0.01	0.00	0.00	0.00	0.00	0.06	2

A169	498	0.76	0.00	0.01	0.00	0.04	0.00	0.13	0.00	0.01	0.02	0.00	0.00	0.00	0.01	2
A170	328	0.76	0.00	0.01	0.00	0.04	0.00	0.13	0.00	0.02	0.03	0.00	0.00	0.00	0.01	2
A171	82	0.50	0.00	0.00	0.00	0.04	0.00	0.18	0.00	0.07	0.15	0.00	0.00	0.00	0.06	2
A175	18	1.00	0.00	0.00	0.00	0.00	0.00	0.00	0.00	0.00	0.00	0.00	0.00	0.00	0.00	2
A179	19	0.52	0.00	0.00	0.00	0.05	0.00	0.42	0.00	0.01	0.00	0.00	0.00	0.00	0.00	3
A181	23	0.48	0.00	0.00	0.00	0.05	0.02	0.38	0.00	0.05	0.00	0.00	0.00	0.00	0.00	3
A182	0.5	0.00	0.00	0.00	0.00	0.00	0.00	0.00	0.00	1.00	0.00	0.00	0.00	0.00	0.00	1
A184	56	0.94	0.00	0.00	0.00	0.02	0.00	0.04	0.00	0.00	0.00	0.00	0.00	0.00	0.00	2
A188	74	0.26	0.00	0.00	0.00	0.00	0.00	0.57	0.00	0.16	0.00	0.00	0.00	0.00	0.01	3
A190	17	0.00	0.00	0.00	0.00	0.00	0.00	0.00	0.00	1.00	0.00	0.00	0.00	0.00	0.00	1
A191	200	0.92	0.00	0.00	0.00	0.01	0.00	0.04	0.00	0.00	0.00	0.00	0.00	0.00	0.02	2
A192	22	0.97	0.00	0.00	0.00	0.00	0.00	0.00	0.00	0.03	0.00	0.00	0.00	0.00	0.00	2
A197	535	0.04	0.01	0.00	0.17	0.09	0.39	0.02	0.11	0.01	0.00	0.00	0.00	0.00	0.16	3
A200	48	0.93	0.00	0.00	0.00	0.01	0.00	0.05	0.00	0.01	0.01	0.00	0.00	0.00	0.00	2
A203	386	0.00	0.01	0.00	0.20	0.09	0.42	0.01	0.14	0.00	0.01	0.00	0.00	0.00	0.11	3
A204	852	0.00	0.00	0.00	0.18	0.13	0.21	0.01	0.38	0.01	0.00	0.00	0.00	0.00	0.07	3
A205	417	0.04	0.01	0.00	0.09	0.11	0.34	0.00	0.31	0.00	0.00	0.00	0.00	0.00	0.11	3
A207	257	0.00	0.00	0.02	0.00	0.00	0.03	0.00	0.00	0.94	0.00	0.00	0.00	0.00	0.00	1
A209	168	0.54	0.00	0.04	0.01	0.00	0.00	0.36	0.00	0.04	0.00	0.00	0.00	0.00	0.00	2
A210	163	0.00	0.00	0.02	0.00	0.00	0.02	0.00	0.00	0.95	0.00	0.00	0.00	0.00	0.00	1
A211	204	0.00	0.00	0.06	0.00	0.00	0.02	0.00	0.00	0.93	0.00	0.00	0.00	0.00	0.00	1
A212	191	0.00	0.00	0.03	0.00	0.00	0.02	0.00	0.00	0.95	0.00	0.00	0.00	0.00	0.00	1
A215	47	0.00	0.00	0.00	0.08	0.21	0.30	0.00	0.28	0.00	0.00	0.00	0.00	0.00	0.13	3
A217	3.2	0.00	0.00	0.00	0.00	0.00	0.00	0.68	0.00	0.09	0.00	0.00	0.00	0.00	0.23	3
A218	1377	0.00	0.01	0.00	0.16	0.12	0.43	0.01	0.11	0.00	0.01	0.00	0.00	0.00	0.14	3
A219	206	0.00	0.00	0.00	0.14	0.25	0.16	0.03	0.37	0.01	0.00	0.00	0.00	0.00	0.04	3
A225	40	0.87	0.00	0.00	0.00	0.03	0.00	0.08	0.00	0.01	0.00	0.00	0.00	0.00	0.00	2
U9	553	0.08	0.01	0.00	0.71	0.00	0.09	0.02	0.02	0.00	0.00	0.00	0.00	0.07	0.00	3
U11	465	0.00	0.01	0.00	0.77	0.00	0.20	0.00	0.02	0.00	0.00	0.00	0.00	0.00	0.00	3
U18	1018	0.92	0.00	0.00	0.03	0.00	0.00	0.04	0.00	0.00	0.00	0.00	0.00	0.00	0.00	2
U19	50	0.91	0.00	0.00	0.00	0.00	0.00	0.06	0.00	0.03	0.00	0.00	0.00	0.00	0.00	2
U3	2040	0.79	0.00	0.00	0.00	0.02	0.00	0.14	0.00	0.01	0.01	0.00	0.00	0.00	0.02	2
U4	2180	0.79	0.00	0.00	0.01	0.02	0.00	0.15	0.00	0.01	0.01	0.00	0.00	0.00	0.01	2
U5	6632	0.87	0.00	0.00	0.00	0.01	0.00	0.09	0.00	0.01	0.00	0.00	0.00	0.00	0.00	2
U6	406	0.00	0.01	0.00	0.09	0.12	0.36	0.01	0.33	0.02	0.01	0.00	0.00	0.00	0.06	3
U7	697	0.83	0.00	0.02	0.01	0.03	0.01	0.07	0.00	0.03	0.00	0.00	0.00	0.00	0.00	2
U8	2053	0.82	0.00	0.01	0.00	0.01	0.00	0.13	0.00	0.01	0.01	0.00	0.00	0.00	0.01	2
U10	415	0.00	0.02	0.01	0.11	0.09	0.42	0.03	0.18	0.02	0.03	0.00	0.00	0.00	0.09	3
U14	14029	0.83	0.00	0.01	0.01	0.03	0.00	0.09	0.00	0.01	0.00	0.00	0.00	0.00	0.01	2
U15	38804	0.77	0.00	0.00	0.01	0.01	0.00	0.16	0.00	0.01	0.00	0.00	0.00	0.00	0.03	2
U16	2570	0.86	0.00	0.00	0.00	0.01	0.01	0.09	0.00	0.01	0.00	0.00	0.00	0.00	0.01	2
U17	4241	0.80	0.00	0.02	0.01	0.02	0.00	0.10	0.00	0.03	0.00	0.00	0.00	0.00	0.01	2
C1	20	0.00	0.00	0.00	0.00	0.00	0.02	0.00	0.00	0.98	0.00	0.00	0.00	0.00	0.00	1
C2	31	0.00	0.00	0.00	0.00	0.00	0.00	0.15	0.05	0.80	0.00	0.00	0.00	0.00	0.00	1
C3	84	0.00	0.00	0.00	0.00	0.00	0.02	0.04	0.00	0.93	0.00	0.00	0.00	0.00	0.01	1
C4	1552	0.31	0.00	0.03	0.01	0.01	0.01	0.41	0.04	0.17	0.00	0.00	0.00	0.00	0.01	3

C5	318	0.16	0.00	0.02	0.00	0.01	0.00	0.53	0.03	0.23	0.00	0.00	0.00	0.00	0.00	3
C6	468	0.22	0.00	0.03	0.00	0.01	0.00	0.49	0.02	0.23	0.00	0.00	0.00	0.00	0.00	3
C7	138	0.00	0.00	0.00	0.00	0.00	0.02	0.01	0.00	0.97	0.00	0.00	0.00	0.00	0.01	1
C8	682	0.30	0.00	0.04	0.00	0.01	0.00	0.45	0.02	0.17	0.00	0.00	0.00	0.00	0.00	3
C9	330	0.22	0.00	0.01	0.00	0.01	0.00	0.60	0.03	0.13	0.00	0.00	0.00	0.00	0.00	3
C10	205	0.00	0.00	0.00	0.00	0.00	0.03	0.04	0.04	0.89	0.00	0.00	0.00	0.00	0.00	1
C11	5166	0.15	0.00	0.02	0.00	0.01	0.00	0.57	0.03	0.21	0.00	0.00	0.00	0.00	0.00	3
C12	833	0.25	0.00	0.02	0.00	0.01	0.00	0.52	0.03	0.16	0.00	0.00	0.00	0.00	0.00	3
C13	333	0.24	0.00	0.00	0.00	0.00	0.00	0.53	0.04	0.18	0.00	0.00	0.00	0.00	0.00	3
C14	493	0.29	0.00	0.02	0.00	0.00	0.00	0.43	0.05	0.20	0.00	0.00	0.00	0.00	0.01	3
C15	303	0.12	0.00	0.01	0.00	0.01	0.00	0.57	0.04	0.25	0.00	0.00	0.00	0.00	0.00	3
C16	5073	0.16	0.00	0.02	0.00	0.01	0.00	0.57	0.02	0.21	0.00	0.00	0.00	0.00	0.00	3
C17	17772	0.33	0.00	0.05	0.00	0.01	0.00	0.41	0.03	0.16	0.00	0.00	0.00	0.00	0.01	3
C18	3741	0.09	0.00	0.01	0.00	0.02	0.00	0.52	0.05	0.30	0.00	0.00	0.00	0.00	0.01	3
C19	2599	0.14	0.00	0.04	0.00	0.02	0.01	0.38	0.05	0.35	0.00	0.00	0.00	0.00	0.01	3
C20	322	0.43	0.00	0.00	0.00	0.03	0.01	0.32	0.07	0.09	0.00	0.00	0.00	0.00	0.05	3
C21	452	0.45	0.00	0.02	0.00	0.02	0.00	0.37	0.06	0.08	0.00	0.00	0.00	0.00	0.01	3
C22	1012	0.40	0.00	0.02	0.00	0.02	0.00	0.36	0.07	0.09	0.00	0.00	0.00	0.00	0.03	3
C23	96	0.39	0.00	0.00	0.00	0.00	0.00	0.38	0.04	0.18	0.00	0.00	0.00	0.00	0.00	3
C24	161	0.36	0.00	0.02	0.00	0.03	0.00	0.39	0.06	0.15	0.00	0.00	0.00	0.00	0.00	3
C25	1786	0.14	0.00	0.03	0.00	0.01	0.01	0.37	0.11	0.25	0.00	0.00	0.00	0.00	0.09	3
C26	859	0.19	0.00	0.03	0.00	0.02	0.01	0.44	0.07	0.19	0.00	0.00	0.00	0.01	0.04	3
C27	328	0.14	0.00	0.03	0.00	0.01	0.01	0.32	0.13	0.33	0.01	0.00	0.00	0.00	0.03	3
C28	452	0.21	0.00	0.03	0.00	0.01	0.01	0.33	0.11	0.24	0.00	0.00	0.00	0.01	0.05	3
C29	1075	0.41	0.00	0.02	0.00	0.01	0.01	0.28	0.07	0.16	0.00	0.00	0.00	0.00	0.04	3
C30	156	0.23	0.00	0.02	0.00	0.00	0.00	0.43	0.05	0.24	0.00	0.00	0.00	0.00	0.03	3
C31	399	0.27	0.00	0.03	0.00	0.01	0.00	0.43	0.04	0.21	0.00	0.00	0.00	0.00	0.01	3
C32	973	0.18	0.00	0.03	0.00	0.01	0.00	0.46	0.03	0.27	0.00	0.00	0.00	0.00	0.01	3
C33	111	0.00	0.00	0.03	0.00	0.00	0.00	0.39	0.05	0.51	0.00	0.00	0.00	0.00	0.03	3
C34	309	0.11	0.00	0.02	0.00	0.00	0.00	0.52	0.06	0.28	0.00	0.00	0.00	0.00	0.01	3
C35	285	0.12	0.00	0.03	0.00	0.01	0.00	0.55	0.04	0.24	0.00	0.00	0.00	0.00	0.01	3
C36	221	0.26	0.00	0.02	0.00	0.01	0.01	0.46	0.05	0.17	0.00	0.00	0.00	0.00	0.02	3
C37	249	0.11	0.00	0.03	0.00	0.00	0.00	0.49	0.07	0.30	0.00	0.00	0.00	0.00	0.01	3
M1	394	0.00	0.01	0.00	0.14	0.38	0.20	0.00	0.05	0.01	0.05	0.00	0.00	0.00	0.16	3
M4	280	0.00	0.00	0.00	0.11	0.17	0.26	0.02	0.10	0.02	0.04	0.00	0.00	0.00	0.27	3
M7	543	0.00	0.00	0.00	0.16	0.22	0.36	0.01	0.03	0.01	0.07	0.00	0.00	0.00	0.13	3
M13	196	0.00	0.00	0.00	0.02	0.23	0.36	0.00	0.11	0.04	0.01	0.00	0.00	0.00	0.22	3
M23	133	0.00	0.00	0.00	0.00	0.33	0.23	0.00	0.09	0.03	0.12	0.00	0.00	0.00	0.21	3
M2	1333	0.02	0.00	0.00	0.21	0.36	0.20	0.01	0.02	0.01	0.05	0.00	0.00	0.03	0.08	3
M5	723	0.00	0.00	0.00	0.23	0.13	0.33	0.04	0.06	0.03	0.05	0.00	0.00	0.00	0.12	3
M8	3680	0.01	0.01	0.00	0.15	0.22	0.30	0.01	0.04	0.01	0.00	0.00	0.00	0.03	0.22	3
M11	846	0.03	0.00	0.00	0.09	0.50	0.09	0.01	0.03	0.01	0.07	0.00	0.00	0.04	0.14	3
M14	935	0.02	0.00	0.00	0.11	0.30	0.23	0.02	0.09	0.02	0.03	0.00	0.00	0.00	0.19	3
M16	446	0.00	0.00	0.00	0.16	0.28	0.25	0.01	0.05	0.01	0.03	0.00	0.00	0.00	0.20	3
M18	947	0.06	0.00	0.00	0.06	0.32	0.12	0.01	0.10	0.01	0.09	0.00	0.00	0.00	0.23	3
M20	483	0.05	0.00	0.00	0.08	0.48	0.13	0.01	0.04	0.01	0.06	0.00	0.00	0.00	0.15	3

M69	393	0.00	0.00	0.00	0.13	0.21	0.38	0.01	0.07	0.02	0.04	0.00	0.00	0.00	0.14	3
M24	1024	0.00	0.01	0.00	0.07	0.21	0.30	0.00	0.06	0.01	0.00	0.00	0.00	0.00	0.33	3
M27	852	0.00	0.00	0.00	0.06	0.08	0.53	0.01	0.08	0.04	0.00	0.00	0.00	0.00	0.20	3
M31	408	0.00	0.01	0.00	0.12	0.12	0.43	0.02	0.08	0.02	0.00	0.00	0.00	0.00	0.21	3
M33	112	0.17	0.00	0.00	0.18	0.05	0.41	0.07	0.08	0.04	0.00	0.00	0.00	0.00	0.00	3
M73	361	0.00	0.01	0.00	0.10	0.13	0.45	0.01	0.08	0.03	0.01	0.00	0.00	0.00	0.19	3
M48	108	0.00	0.00	0.00	0.11	0.03	0.61	0.02	0.12	0.09	0.00	0.00	0.00	0.00	0.01	3
M59	177	0.00	0.00	0.00	0.12	0.03	0.69	0.03	0.06	0.06	0.00	0.00	0.00	0.00	0.01	3
M3	113	0.00	0.00	0.00	0.17	0.49	0.19	0.00	0.04	0.02	0.00	0.00	0.00	0.00	0.08	3
M9	199	0.00	0.00	0.00	0.11	0.40	0.29	0.00	0.05	0.01	0.02	0.00	0.00	0.00	0.11	3
M17	107	0.00	0.00	0.00	0.04	0.04	0.73	0.00	0.08	0.05	0.00	0.00	0.00	0.00	0.06	3
M21	61	0.00	0.00	0.00	0.07	0.35	0.42	0.01	0.06	0.02	0.00	0.00	0.00	0.00	0.06	3
M71	30	0.00	0.00	0.00	0.00	0.09	0.67	0.00	0.10	0.05	0.00	0.00	0.00	0.00	0.10	3
M47	97	0.00	0.00	0.00	0.07	0.06	0.78	0.00	0.06	0.04	0.00	0.00	0.00	0.00	0.00	3
M58	125	0.00	0.00	0.00	0.13	0.10	0.45	0.00	0.13	0.04	0.03	0.00	0.00	0.00	0.13	3
	Total	241	241	241	241	241	241	241	241	241	241	241	241	241	241	
	Non-zero	168	31	160	104	201	171	204	151	238	65	113	82	11	193	
	Non-zero %	70%	13%	66%	43%	83%	71%	85%	63%	99%	27%	47%	34%	5%	80%	

Table S3. Summary of statistics (minimum, maximum, mean, median and variance) obtained from log transformed PST toxicity data generated by the five quantitative testing methods (MBA, PreCOX, PCOX, LC-MS/MS and RBA)

Method	Min	Max	Mean	Median	Variance
MBA	5.226	12.631	7.968	7.415	3.475
PreCOX	5.263	12.598	7.684	7.427	3.147
PCOX	3.664	10.716	7.134	7.080	2.111
LCMSMS	4.431	12.907	7.778	7.438	3.170
RBA	5.375	12.510	7.888	7.585	2.961

Table S4. Summary of statistics (minimum, maximum, mean, median and variance) obtained from log transformed PST toxicity data generated by the four quantitative testing methods (MBA, PreCOX, PCOX and LC-MS/MS)

Method	Min	Max	Mean	Median	Variance
MBA	5.226	12.631	8.364	7.762	3.466
PreCOX	5.263	12.598	8.010	7.734	3.079
PCOX	3.664	11.114	7.567	7.615	2.297
LCMSMS	4.431	12.907	8.063	7.816	3.152

Figure S1. Bar charts illustrating sample types associated with each toxin profile cluster, in relation to a) country of origin and b) shellfish species. Arg = Argentina, Mex = Mexico, Uru = Uruguay; M = mussel, Cl = Clam, Sc = scallop, G = gastropod, Ge = geoduck, O = oyster, Misc = Miscellaneous

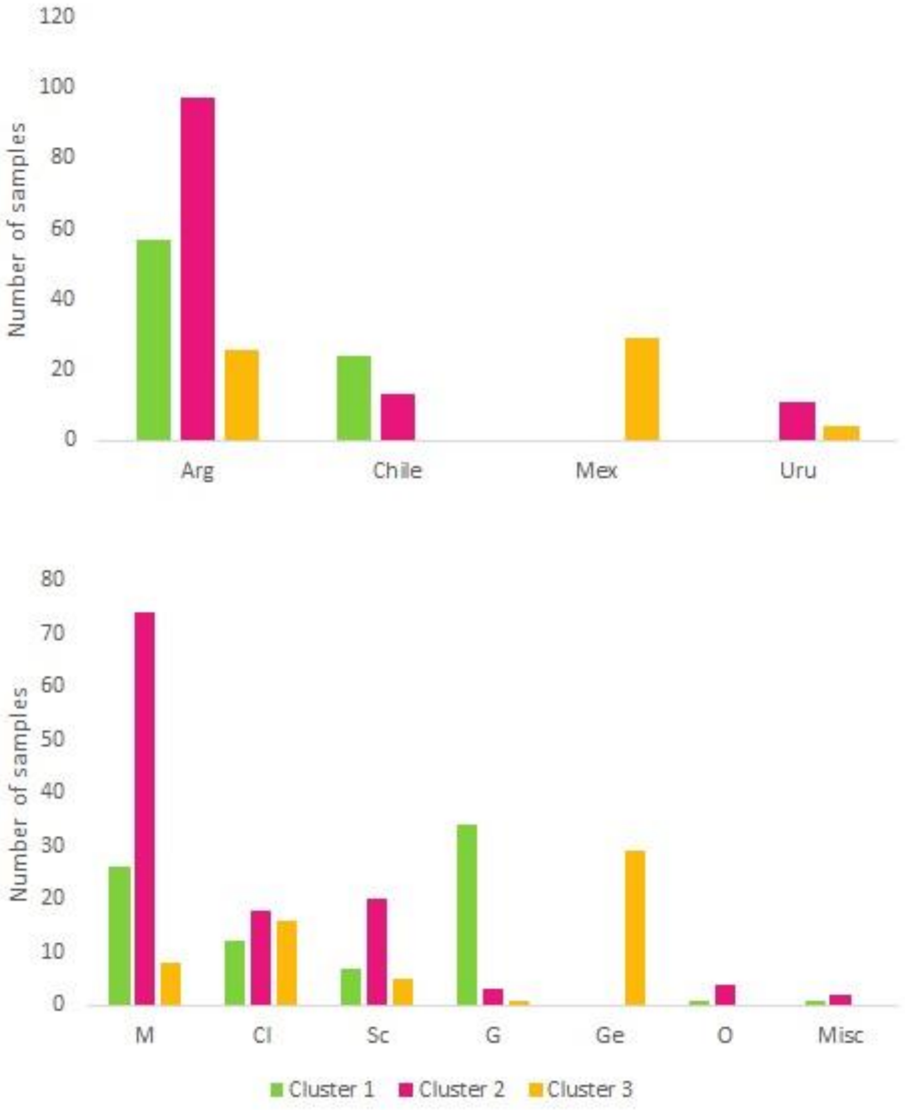

Supplement: Supplementary file 1 [file marinedrugs-18-00616-s001.pdf]
